# Activity of distinct growth factor receptor network components in breast tumors uncovers two biologically relevant subtypes

**DOI:** 10.1186/s13073-017-0429-x

**Published:** 2017-04-26

**Authors:** Mumtahena Rahman, Shelley M. MacNeil, David F. Jenkins, Gajendra Shrestha, Sydney R. Wyatt, Jasmine A. McQuerry, Stephen R. Piccolo, Laura M. Heiser, Joe W. Gray, W. Evan Johnson, Andrea H. Bild

**Affiliations:** 1 Department of Pharmacology and Toxicology, University of Utah, 30 S 2000 E, Salt Lake City, UT 84108 USA; 2 Department of Biomedical Informatics, University of Utah, Salt Lake City, UT USA; 3 Department of Oncological Sciences, University of Utah, Salt Lake City, UT USA; 4 Division of Computational Biomedicine, Boston University School of Medicine, Boston, MA USA; 5 Department of Biomedical Engineering, Center for Spatial Systems Biomedicine, Knight Cancer Institute, Oregon Health and Sciences University, Portland, OR USA; 6 Department of Biology, Brigham Young University, Provo, UT USA

**Keywords:** Breast cancer, Gene expression signatures, Cancer phenotypes, Growth factor receptor network, Genomics, Targeted therapy

## Abstract

**Background:**

The growth factor receptor network (GFRN) plays a significant role in driving key oncogenic processes. However, assessment of global GFRN activity is challenging due to complex crosstalk among GFRN components, or *pathways*, and the inability to study complex signaling networks in patient tumors. Here, pathway-specific genomic signatures were used to interrogate GFRN activity in breast tumors and the consequent phenotypic impact of GRFN activity patterns.

**Methods:**

Novel pathway signatures were generated in human primary mammary epithelial cells by overexpressing key genes from GFRN pathways (*HER2*, *IGF1R*, *AKT1*, *EGFR*, *KRAS* (*G12V*), *RAF1*, *BAD*). The pathway analysis toolkit Adaptive Signature Selection and InteGratioN (ASSIGN) was used to estimate pathway activity for GFRN components in 1119 breast tumors from The Cancer Genome Atlas (TCGA) and across 55 breast cancer cell lines from the Integrative Cancer Biology Program (ICBP43). These signatures were investigated for their relationship to pro- and anti-apoptotic protein expression and drug response in breast cancer cell lines.

**Results:**

Application of these signatures to breast tumor gene expression data identified two novel discrete phenotypes characterized by concordant, aberrant activation of either the HER2, IGF1R, and AKT pathways (“the survival phenotype”) or the EGFR, KRAS (G12V), RAF1, and BAD pathways (“the growth phenotype”). These phenotypes described a significant amount of the variability in the total expression data across breast cancer tumors and characterized distinctive patterns in apoptosis evasion and drug response. The growth phenotype expressed lower levels of BIM and higher levels of MCL-1 proteins. Further, the growth phenotype was more sensitive to common chemotherapies and targeted therapies directed at EGFR and MEK. Alternatively, the survival phenotype was more sensitive to drugs inhibiting HER2, PI3K, AKT, and mTOR, but more resistant to chemotherapies.

**Conclusions:**

Gene expression profiling revealed a bifurcation pattern in GFRN activity represented by two discrete phenotypes. These phenotypes correlate to unique mechanisms of apoptosis and drug response and have the potential of pinpointing targetable aberration(s) for more effective breast cancer treatments.

**Electronic supplementary material:**

The online version of this article (doi:10.1186/s13073-017-0429-x) contains supplementary material, which is available to authorized users.

## Background

Breast cancer remains one of the leading causes of cancer-related death in women [1]. It is well established that growth factor receptors and their downstream signaling pathways, contribute to breast cancer proliferation, survival, and metastasis [2, 3]. Molecular aberrations can occur in various growth factor receptor network (GFRN) members and have been described in breast cancer [4–6]. These findings have paved the way for GFRN-targeted treatments which are currently approved for use and being evaluated in various stages of clinical development and in clinical trials [7, 8]. Although these treatments do hold promise, relatively few data are available on the cooperativity and diversity of complicated GFRN signaling in actual breast tumors. Additionally, assessing GFRN activity in patient tumors is extremely challenging due to the lack of methods capable of measuring signaling events in tumors. Drug selection is often guided by expression of protein biomarkers, and drug resistance often develops due to compensation by interacting pathways within the GFRN [9, 10]. Therefore, there is a strong need to develop better methods for measuring and understanding GFRN signaling events in breast tumors in order to deliver the most effective treatment regimens and combat drug resistance [2, 9, 11].

Growth factor receptors, such as epidermal growth factor receptor 1 (EGFR), human epidermal growth factor receptor 2 (HER2), and insulin-like growth factor 1 receptor (IGF1R), are key regulatory nodes of the GFRN and are often aberrantly activated across breast cancer subtypes [6, 12, 13]. Approximately 15–30% of breast cancer patients are diagnosed with HER2-positive breast cancer, which is characterized by amplification of HER2 [12]. *EGFR* amplifications occur in 25% of all triple-negative breast cancer (TNBC) patients and are often associated with poor outcomes [6, 8, 14]. High IGF1R activity occurs in up to 50% of breast tumors and is seen across all breast cancer subtypes [13]. These receptors can activate downstream oncogenic growth cascades such as the phosphoinositide 3-kinase (PI3K) and mitogen-activated protein kinase (MAPK) pathways, forming a complex, interconnected, and dynamic signaling network [2, 8]. Activation of PI3K by growth factor receptors triggers the PI3K/AKT/mammalian target of rapamycin (mTOR) pathway, leading to cell proliferation, metabolic changes, and cell survival [15–17]. In the MAPK pathway, following growth factor receptor activation, RAS becomes activated followed by activation of RAF1, MEK, and ERK, leading to transcriptional changes that impact cellular proliferation, motility, and evasion of apoptosis [6, 8, 18, 19]. Both the PI3K and MAPK pathways contribute to tumor progression by disrupting the balance of pro- and anti-apoptotic proteins of the BCL-2 protein family in the mitochondrial (also known as intrinsic) pathway of apoptosis [20, 21]. Particular GFRN members can upregulate anti-apoptotic proteins such as BCL-2, BCL-XL, and MCL-1 and downregulate pro-apoptotic proteins such as BAD, BAX, and BIM, all of which contribute to apoptosis evasion and resistance to cancer treatments in patients [22–29]. ERBB receptor tyrosine kinases, such as EGFR and HER2, have a great deal of overlap in the downstream pathways they activate; however, individual ERBB receptors have the capability to preferentially bind particular downstream signaling molecules [30, 31]. Furthermore, preclinical studies have shown that EGFR− and HER2-driven cancers show differential response to targeted therapies. *EGFR* mutant cancers are less responsive to single-agent PI3K/AKT inhibitors in comparison to HER2-amplified cancers and require the inhibition of both the PI3K and MEK pathways [32]. These suggest that ERBB proteins can couple to distinct signaling pathways and invoke non-redundant physiological effects, which warrants for specificity for the different GFRN components. Therefore, an accurate assessment of global GFRN activity is pivotal for selecting targeted treatment strategies that consider the diversity of growth and cell survival mechanisms in breast cancer patients.

Despite advances in the cellular and molecular characterization of breast cancer, effective personalized breast cancer treatment remains elusive. Immunohistochemical and gene expression profiling-defined breast cancer molecular classification has advanced our understanding of breast cancer prognosis, treatment, and improved survival. Currently, breast cancers are stratified into different clinical subtypes in order to determine specific treatments, and several breast cancer subtyping approaches are currently available. For example, fluorescence in situ hybridization (FISH) or immunohistochemistry (IHC) techniques are often used to determine clinical subtypes based on common receptor protein alterations such as estrogen (ER), progesterone (PR), and HER2 receptor amplification [7, 33]. Additionally, Ki-67 (proliferation marker), CK 5/6 (cytokeratin marker), EGFR, androgen receptor (AR), and p53 (apoptosis marker) are used as biomarkers to further classify breast cancer using IHC methods. Although helpful, IHC methods are often subjected to bias due to tissue handling, fixation, antibody sources, and need for physical evaluation by pathologists [34, 35]. More recently, Perou [14, 36] and Sørlie et al. [37] proposed five “intrinsic subtypes” that have shown utility in guiding therapy by leveraging gene expression data, differences in clinical outcomes, and responses to neoadjuvant chemotherapy [7, 38]. Further, evaluation of gene expression has led to the proposition of several additional subtypes, including claudin-low, molecular apocrine, and a novel luminal-like subtype [39–44]. While molecular subtypes continue to emerge, routine use of such subtypes in clinical settings is not sensitive and specific due to some critical limitations. For example, tumors of the same clinical or intrinsic subtype can show differences in growth, survival, and response to therapies [45], and clinical and intrinsic subtypes are sometimes discrepant [46]. Approximately one-third of HER2+ tumors are not classified as the HER2-enriched intrinsic subtype and up to 25% of clinically characterized ER+ tumors are not classified as the luminal intrinsic subtype [36]. While IHC methods are single protein based, intrinsic subtypes are fundamentally empirical and do not focus on distinct biological properties. Thus, both IHC and intrinsic subtypes fail to recapitulate the biological heterogeneity within each subtype [47]. Recent studies highlight the discordance between the IHC and intrinsic subtypes, which calls for additional work [47, 48]. To address these challenges, pathway-level subtyping may provide complementary information for determining therapeutic targets. For example, identification of specific aberrant pathways within the triple negative and basal-like subtypes may help to explain additional heterogeneity and better target these subtypes pharmacologically [49]. Here, breast cancer inter-tumor heterogeneity was explored in terms of GFRN activity for its well-known role in growth, evasion of apoptosis, and drug response.

**Fig. 1:**
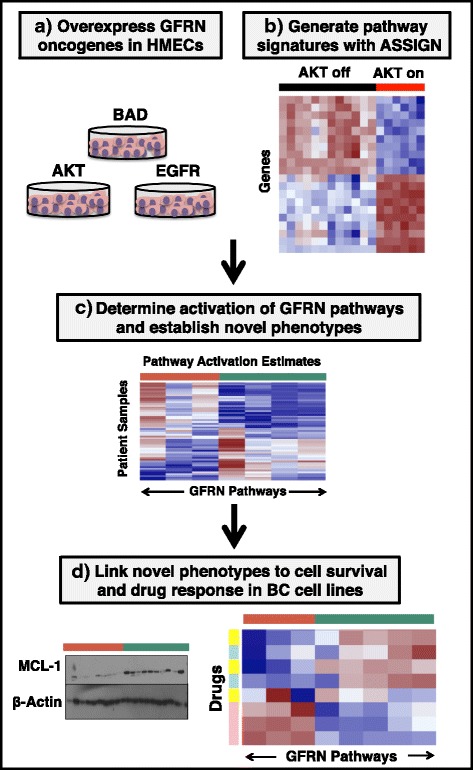
High-level overview for probing growth factor receptor networks in breast cancer. **a** Overexpression of growth factor receptor network (*GFRN*) genes in human mammary epithelial cells (*HMECs*): AKT, BAD, EGFR, HER2, IGF1R, RAF1, and KRAS (G12V). **b** Generation of RNA-sequencing data from HMECs overexpressing GFRN genes and signature generation using ASSIGN. **c** Determination of GFRN pathways activation across TCGA breast tumors and ICBP breast cancer cell lines and identification of novel phenotypes based on GFRN activity. **d** Linking novel phenotypes to survival and drug response mechanisms in biochemical and drug response assay

While biochemical measurement of pathway activity is challenging in human tumors due to limited tissue availability and instability of specific proteins, patterns of activity across multiple genes—or gene expression *signatures*—can be used as surrogates for pathway activation in tumors and to model biological phenotypes [50–54]. Pathway activation has been used to predict drug response to targeted therapies in cell lines [52, 54, 55], but to the best of our knowledge, this is the first study which measures activity of seven GFRN members concurrently at the pathway level in patient samples. In this study, 1119 breast tumors were profiled for GFRN activity across The Cancer Genome Atlas (TCGA) and across 55 breast cancer cell lines from the Integrative Cancer Biology Program (ICBP43) [56, 57] (Fig. 1). Pathway activity was estimated in samples using novel GFRN gene expression signatures for the HER2, IGF1R, AKT, EGFR, KRAS (G12V mutation), RAF1, and BAD pathways. These GFRN signatures were generated by performing sequencing on RNA collected from primary human mammary epithelial cells (HMECs) overexpressing *HER2*, *IGF1R*, *AKT1*, *EGFR*, *KRAS* (*G12V*), *RAF1*, or *BAD* for 18–36 h. These signatures capture early transcriptional events, which occur shortly after oncogene activation, and represent the transcriptional profile of pathway activation, and not of a transformed cell.

Using the pathway analysis toolkit Adaptive Signature Selection and InteGratioN (ASSIGN), the signatures were projected onto each breast cancer data set and uncovered two discrete patterns of GFRN activity [58]. One pattern was characterized by concurrent activation of the HER2, IGF1R, and AKT pathways, and another was characterized by concurrent activation of the EGFR, KRAS, RAF1, and BAD pathways. Typically, when one set of pathways was active, the other set was inactive, indicating that each sample tends to have a dominant GFRN phenotype. Pathways activation of HER2, IGF1R, and AKT was nicknamed the “survival phenotype” and activation of EGFR, KRAS, RAF1, and BAD as the “growth phenotype”. These names were chosen for simplicity and based on the known role of AKT signaling in cancer cell survival and the known role of EGFR/RAS signaling in cellular growth [59, 60]. Importantly, genomic pathway activity corresponded to apoptotic phenotypes. The growth phenotype showed upregulation of anti-apoptotic protein MCL-1 and downregulation of pro-apoptotic protein BIM as a mechanism of escaping apoptosis. Additional subgroups were also identified within each phenotype, including HER2 high and HER2 low activity groups within the survival phenotype and BAD high and BAD low activity groups within the growth phenotype. These discrete subgroups displayed differences in response to targeted therapies and chemotherapies. Therefore, these phenotypes can serve as surrogates for GFRN activity that capture significant variability in the gene expression data, differentiate survival mechanisms, and correlate to drug response significantly. A major component of the heterogeneity found across tumor expression data was contributed by GFRN signaling and was independent of ER, PR, and HER2 status compared to intrinsic subtypes. Additionally, a unique aspect is that GFRN activity explained the data in a biologically meaningful way. For example, while intrinsic subtyping approaches are based on empirical patterns of gene expression and do not necessarily represent a biological process, the subgrouping approach represents aberrant activity in specific GFRN pathway signaling. Therefore, pathway-based phenotypes and subgroups have the potential to complement existing methods and identify biologically and clinically relevant patterns in tumors. Taken together, pathway signatures not only aid in assessing general pathway activity patterns in a biologically relevant way, but also show promise to select better treatment targets for breast cancer patients.

## Methods

### Overexpression of genes of interest in human mammary epithelial cells

In order to create gene expression signatures representative of pathway activation, GFRN oncogenes were overexpressed in HMECs. HMECs from a non-cancer-related breast reduction surgery performed at the University of Utah were isolated and cultured according to previously published protocols [61]. Cells were grown in serum-free mammary epithelial basal medium (MEBM) plus the addition of a “bullet kit” (Lonza) and supplemented with 5 mg/ml transferrin and 10^−5^ M isoproterenol at 5% CO_2_. Cells were brought to quiescence by growth in low serum conditions (0.25% MEBM + bullet kit, no EGF) for 36 h. Cells were infected with recombinant adenovirus (at 500 MOI) expressing either human oncogenes *AKT1*, *IGF1R*, *BAD*, *HER2*, *KRAS* (*G12V*), and *RAF1* or *GFP* control (Additional file 1: Figure S1). Cells were incubated with virus for 18 h except for *KRAS* (*G12V*), which was incubated for 36 h. The adenoviral expression systems invoke transient gene expression changes, which allow us to capture the early transcriptional events of each oncogene, as opposed to the transcriptional profile of a transformed cell. Recombinant adenoviruses were amplified and concentrations were determined using previously published protocols [62]. All viruses were obtained from Vector Biolabs, except *RAF1* (Cell Biolabs) and *EGFR* (gift from Duke University).

### Western blot analysis for expression of growth factor proteins in HMECs and apoptotic proteins in breast cancer cell lines

Proteins from HMECs and following cell lines were extracted: HCC3153, HCC1395, ZR75B, HCC1569, HCC2218, SKBR3, LY2, SUM52PE, ZR7530, MDAMB361, AU565, BT474, BT483, CAMA1, HCC1419, HCC1428, MCF7, MDAMB175, T47D, ZR751, HCC1954, JIMT1, BT549, HCC1143, HCC1806, HCC1937, HCC38, HCC70, HS578T, and MDAMB213 (Additional file 2: Sheet 1). To collect protein, cells were washed with PBS, scraped on ice into PBS, pelleted by centrifugation, lysed in lysis buffer for 15 minutes (50 mM Tris (pH 8.0), 140 mM NaCl, 5 mM EDTA, 1% TritionX-100, 0.1% SDS, protease cocktail (Sigma), phosphatase inhibitors cocktails 2 and 3 (Sigma), and centrifuged at 13,000 × g for 15 minutes. Protein quantification of lysates was determined using a BCA assay (Pierce). Electrophoresis was performed on a 8–12% Tris-HCl polyacrylamide gel (BioRad) for HMEC Western blots and 18% Criterion TGX Tris/Glycine gels (BioRad) for apoptotic protein western blots. Proteins were then transferred to a PVDF membrane using the iBlot® 2 Dry Blotting System (Thermo Fisher Scientific). Membranes were blocked for 1 h with SuperBlock™ (Thermo Fisher Scientific) and probed with the following primary antibodies: AKT (#9272), pAKT (#13038), BAD (#9292), EGFR (#4267), pEGFR (#2234), HER2 (#2165), pHER2 (#2244), IGF1R (#3027), pIGF1R (#3021), KRAS (sc-30), pMEK (#9154), p-cRAF (#9427), GAPDH (#5174), and β-tubulin (#2146). Of note, pAKT ran higher than expected due to AKT myristoylation. Breast cancer cell line lysates were probed with the following: MCL-1 (#5453), BIM (#2933), and B-actin (#3700). All antibodies were obtained from Cell Signaling Technology, besides KRAS, which was obtained from Santa Cruz.

### Dose response assay

Cell lines were plated at 2000 cells per well in 384 well plates for 24 h at 37 °C. Detailed information on the cell lines and their growth conditions is provided in Additional file 2: Sheet 1. All cell lines were obtained from American Type Culture Collection (ATCC). Drugs were diluted to six doses in media containing 5% FBS (Gibco/Life technologies) and 1% anti–anti (Gibco/Life technologies). Erlotinib, trametinib, UMI-77, obatoclax, doxorubicin, and neratinib were purchased from Selleckchem, and bafilomycin and AKT1/2 inhibitor were from Sigma-Aldrich. Drugs were dissolved in 100% DMSO and stored at −80 °C. Detailed information on drug doses is provided in Additional file 2: Sheet 2. Cell viability and growth was measured using CellTiter-Glo (Promega) 72 h post-treatment. All treatment doses were performed in four replicates. The Drug Discovery Core Facility, a part of the Health Sciences Cores at the University of Utah, performed the dose response assay. EC50s (concentration of each drug that provides half of the maximum response) were determined and converted to drug sensitivity values defined as the negative log of the EC50s (−logEC50) (Additional file 2: Sheet 3). EC50 values were calculated from dose response data by plotting in GraphPad Prism 4 and using the equation *Y* = 1/(1 + 10ˆ((logEC50 − *X*) × HillSlope)) with a variable slope (*Y*_min_ = 0 and *Y*_max_ = 1).

### RNA preparation and RNA sequencing

After transfection with adenovirus and Western blot validation, cells were pelleted, washed in PBS, and stored in RNAlater (Ambion). Cells were then DNase treated, and RNA was extracted using the RNeasy kit (Qiagen). RNA replicates were generated for each overexpressed gene: six each for *AKT*, *BAD*, *IGF1R*, and *RAF1*; five for *HER2*; and 12 for *GFP* control (Gene Expression Omnibus (GEO) accession GSE83083). Additionally, 9 replicates of each of *KRAS* and *GFP* control were generated (GEO accession GSE83083). The *EGFR* signature and its corresponding *GFP* control were previously generated with six replicates of each (GEO accession GSE59765). RNA concentration was determined with a Nanodrop (ND-1000). cDNA libraries were prepared from extracted RNA using the Illumina Stranded TruSeq protocol (Illumina). cDNA libraries were sequenced at Oregon Health and Sciences University using the Illumina HiSeq 2000 sequencing platform with six samples per lane. Single-end reads of 101 base pairs were generated.

### Gene expression data processing, normalization, and datasets

The *Rsubread* R package (version 1.14.2) was used to align and summarize RNA-seq reads to the UCSC hg19 reference genome and annotations [63, 64]. All RNA-seq data in this study, including HMEC overexpression data (GSE83083, GSE59765), TCGA breast cancer data (GSE62944), and ICBP breast cancer RNA-Seq dataset (GSE48213), were processed and normalized using a pipeline that can be found at https://github.com/srp33/TCGA_RNASeq_Clinical [60, 65].

### Generation of gene expression signatures

Adaptive Signature Selection and InteGratioN (ASSIGN; version 1.9.1), a semi-supervised pathway profiling toolkit, was used to generate gene expression signatures. A formal definition of the ASSIGN model and software implementation was reported previously [58]. RNA-Seq data from HMECs overexpressing GFP control were compared to HMECs overexpressing *AKT1*, *IGF1R*, *BAD*, *HER2*, *KRAS (G12V)*, *RAF1*, and *EGFR.* ASSIGN uses a Bayesian variable approach to select genes with the highest weights and signal strengths, indicating differential expression. These genes represent oncogenic signatures (Additional file 1: Figure S2).

### Gene set enrichment analysis on RNA-Seq signatures

The R package Gene Set Variation Analysis for microarray and RNA-Seq data (GSVA; version 1.22.0), a non-parametric, unsupervised method for estimating variation of gene set enrichments in gene expression data, was used to perform this gene set enrichment analysis [66]. GSVA was downloaded from Bioconductor (3.4). RNA-Seq data from HMECs overexpressing *GFP* (control), *AKT1*, *IGF1R*, *BAD*, *HER2*, *KRAS(G12V)*, *RAF1*, and *EGFR* was used as input for the GSVA algorithm. The following gene sets were used and downloaded from the Molecular Signatures Database (http://software.broadinstitute.org/gsea/downloads.jsp) [67]; 1320 gene sets from the C2: canonical pathways collection (*c2.cp.v5.2.symbols.gmt*) and 50 gene sets from the hallmarks collection (*h.all.v5.2.symbols.gmt*). The following GSVA parameters were used: minimum gene set size = 10, maximum gene set size = 500, verbose = TRUE, rnaseq = TRUE, and method = “ssgsea”. GSVA returns a matrix containing enrichment scores for each sample and gene. The R package limma (version 3.30.2) [68] was used to perform a differential expression analysis between each overexpressed gene sample and its respective GFP control sample. The full results from the gene set enrichment analysis can be found in Additional file 3.

### Batch adjustment and estimation of pathway activity in ICBP and TCGA BRCA patient samples

HMEC oncogenic signatures (training data) were applied to 55 ICBP breast cancer cells and 1119 TCGA breast cancer patient gene expression datasets (test data) to estimate pathway activation status. To avoid confounding batch effects within and between the training and test data, the data were adjusted for batch effects. First, in order to visualize batch effects in the data a principal component analysis (PCA) was performed on the training (HMEC overexpression RNA-Seq) data. The training data were sequenced separately in three batches, and significant batch effects were observed. Batch effects were adjusted using the *ComBat* function from the R package sva (version 3.21.1) [65, 69]. ComBat was run using the reference-batch option, which adjusts the data to match an indicated batch. The sequencing batch containing AKT1, IGF1R, BAD, HER2, and RAF1 was selected as the reference batch. A model-matrix indicating which pathway was associated with each training replicate was also included. After the first batch adjustment, PCA was performed on the adjusted training data and the test data (ICBP breast cancer cell lines or TCGA breast tumors). Significant batch effects were identified between the training and test data and performed a second round of ComBat adjustment, using the training data as the reference batch. After the second batch adjustment, PCA was performed to confirm the resolution of the batch effect. Additionally, background baseline gene expression differences were adjusted between oncogenic signatures and test samples (ICBP cell lines and TCGA patient data) using ASSIGN’s adaptive background parameter. The variation in magnitude and direction of signature-relevant gene expression between oncogenic signature training samples and test samples was adjusted using ASSIGN’s adaptive signature parameter. The model specification options for all analyses are listed in Additional file 1: Table S1. Default ASSIGN settings were used for all other parameters.

### Optimization of single-pathway estimates in ICBP cell line and TCGA BRCA patient data

To determine the optimum number of genes for each oncogenic signature, signatures with gene list lengths from 25 to 500 genes, in 25 gene increments, were generated using ASSIGN’s single pathway settings. By default, ASSIGN chooses gene lists that contain an equal number of genes that have increased or decreased expression with pathway activation. ASSIGN also allows a specific gene to be anchored in the signature, making sure that the gene is always included in the signature, even if it is not chosen during gene selection or if it is removed from the signature after Monte Carlo simulation. Anchor genes were chosen based on the oncogene overexpressed in each signature. Pathway predictions generated by ASSIGN are represented as values from zero to one. Values of zero represent no pathway activity and values of one represent high pathway activity. For all the signatures that passed internal leave-one-out cross-validation, pathway estimates were included for further validation in proteomics, mutation, and gene expression. To determine optimal signature gene list lengths and evaluate the robustness of the generated signatures, pathway activation estimates from ICBP and TCGA were correlated with proteins that reflect downstream pathway activation from corresponding ICBP and TCGA RPPA data as a measurement of protein quantity [70, 71]. Significant correlations were found between pathway activation estimates for all GFRN signatures and appropriate downstream pathway proteins [13, 72–74] (Additional file 1: Table S1). Mutation-based analysis was performed using *t*-tests between patient groups based on mutation status in oncogenic proteins. For example, TCGA mutation data were analyzed and higher AKT activation and lower BAD activation estimates were found in patients with *PI3KCA* mutations (Additional file 1: Figures S3a, b) and higher HER2 pathway activation estimates were found in HER2-positive tumors (Additional file 1: Figure S3c). In gene expression data, higher pathway activity for AKT, EGFR, IGF1R, and RAF1 in TCGA samples classified as “high” expressing using percentiles from TCGA RNA-Seq dataset for their respective genes *AKT1*, *EGFR*, *IGF1R*, and *RAF1* were found (Additional file 1: Figure S3d–g). Samples with 90th percentile or higher expression were considered “high”, 10th percentile or lower “low”, and 10th to 90th percentile “intermediate” expressing samples for AKT1, EGFR, and RAF1. For IGF1R validation, samples with 80th percentile or higher IGF1R expression were considered “high”, 20th percentile or lower “low”, and 20th to 80th percentile “intermediate” expressing samples. Finally, pairwise Spearman correlation values and calculated *p* values between pathway predictions and corresponding TCGA reverse phase protein array (RPPA) data were used to determine which gene numbers gave the best correlations. The HER2 and AKT signatures performed better with fewer genes. Therefore, 5, 10, 15, and 20 gene signatures for HER2 and AKT were generated. Significant correlations were seen between pathway estimates and RPPA protein scores. For example, AKT pathway activation estimates were significantly correlated with AKT, PDK1, and phosphorylated-PDK1 protein levels in both ICBP and TCGA (*p* values <0.0001) samples. Due to the lack of proteins available to validate the BAD signature, negative correlations between BAD pathway estimates and AKT protein based on the knowledge that activation of AKT leads to BAD inhibition were used [23]. The optimized gene list was the list that gave the best average correlation in the expected direction for the RPPA data correlated with each pathway in TCGA data and was significant both in ICBP and TCGA data, with an ICBP correlation of at least 0.3 and a maximum gene list length of 300 genes. Additional file 4 includes a gene list of optimum gene numbers determined for each signature. Additional file 5 contains scaled ASSIGN pathway activity predictions for each of the seven optimized pathways in TCGA and ICBP.

### Software implementation of pathway activity prediction with generated signatures

The signatures presented here have been included in the latest version of the ASSIGN package (version 1.11.3) so that pathway activity prediction can be easily performed on other datasets. Because the gene list length can affect the results of ASSIGN analysis, the signatures can be used in their original form, or the gene list lengths can be optimized based on maximizing correlations between ASSIGN activity predictions and a set of variables, such as RPPA data.

### Determination of growth factor phenotypes in ICBP and TCGA

Cell lines from ICBP, patient tumors from TCGA, and breast cancer cell lines for in vitro experiments were classified as either the survival or growth phenotype by calculating the mean of scaled pathway activation values for HER, IGF1R, and AKT for the survival phenotype and the mean of scaled pathway activation values for BAD, EGFR, KRAS, and RAF1 for the growth phenotype. Each sample was classified as either survival or growth phenotype based on which phenotype had the highest mean (Additional file 5).

### Identification of additional drug response heterogeneity within growth factor phenotypes

To classify samples into subgroups within the growth factor phenotypes that corresponded to high and low HER2 activity within the survival phenotype and high and low BAD activity within the growth phenotype, the R function *kmeans* was used to perform k-means clustering on the scaled pathway activity data for AKT, HER2, BAD, and EGFR pathways with four means and 100 random starts. Additional file 5 contains sample classifications for the ICBP and TCGA data. After classifying samples, *t*-tests were performed using the R function *t.test* on known HER2/AKT/PI3k/mTOR targeting drugs and EGFR/MEK targeting drugs from the drug response assay described above between the cell lines identified as AKT/HER2 high and AKT/HER2 low, and between the cell lines identified as EGFR/BAD high and EGFR/BAD low. *P* values were corrected using a FDR correction and identified drugs that showed a significantly different drug response among the growth factor subgroups. When determining how growth phenotypes and ER, PR, and HER2 status performed in assessing drug response, mean drug response across all available cell lines as the cutoff were used. Cell line drug sensitivity value above this cutoff was considered as “sensitive” and otherwise “resistant”.

### Statistical analyses

The *prcomp* function from the stats R package was used to compute the principal components in TCGA breast cancer patient RNA-Seq data. The Spearman rank-based pairwise correlation method was used for all principal component-based correlations, pathway predictions, and protein correlations. The *cor.test* function from the stats R package was used to calculate *p* values for each correlation [75–77]. Student’s *t*-tests were used to find the differences in principal component values based on IHC-based subtypes and mutation status within GFRN phenotypes; pathway activity based on mutation status and drug; sensitivity differences based on pathway activity, and gene expression boxplots. The *heatmap.2* function from the ggplots R package and the *Heatmap* function from the ComplexHeatmap R package were used for generating pathway activity and pathway activity–drug response correlation heatmaps [78, 79]. The *lm* function from the stats R package was used to model principal component values in TCGA using clinical subtypes, intrinsic subtypes, and GFRN subgroups to determine R^2^ values. Models were compared using the *anova* function from the stats package to determine significance of adding additional features to the models. All analyses were conducted in R and the code is available at https://github.com/mumtahena/GFRN_signatures [80].

## Results

### Two dominant phenotypes in breast cancer patients and cell lines

Gene expression signatures were developed and validated for the following GFRN pathways: AKT, BAD, EGFR, HER2, IGF1R, KRAS (G12V mutation), and RAF1. Signatures were generated in normal human mammary epithelial cells (HMECs) by expressing these genes using recombinant adenoviruses. The control samples received green fluorescent protein (GFP) adenovirus. The overall goal of this approach was to capture the downstream transcriptional events specific for each expressed GFRN gene, or the gene expression signatures, and to use these signatures to estimate pathway activity in cell lines and patient samples. To determine if adenovirus infection led to pathway activation for each overexpressed gene, protein levels of gene products and their downstream targets were measured the using western blotting (Additional file 1: Figure S1). Next, RNA-Seq was performed on multiple replicates of HMECs overexpressing GFRN genes and GFP controls. These data were used to generate pathway-based gene expression signatures for each overexpressed gene using the previously published ASSIGN pathway profiling approach (Additional file 1: Figure S2a–g) [58]. Briefly, ASSIGN prioritized genes that best discriminated GFP control samples from samples overexpressing GFRN genes to generate gene expression signatures. Next, ASSIGN was used to estimate the activation of each GFRN member (AKT, BAD, EGFR, HER2, IGF1R, KRAS (G12V), and RAF1) in 1119 breast cancer patient samples from TCGA and 55 samples from the ICBP panel of breast cancer cell lines. ASSIGN was used to measure highly correlated GFRN pathway activity more accurately in patient samples with signatures generated in HMECs since ASSIGN estimates correlated pathway activities robustly by adapting pathway signatures into specific disease context. The robustness of each pathway signature was validated with (1) leave-one-out cross-validation (LOOCV), (2) relevant reverse phase protein array (RPPA) scores, (3) gene expression data for the overexpressed oncogenes, and (4) mutation data (Additional file 1: Methods, Figure S3, and Table S2). After validating the GFRN signatures, gene set enrichment analysis was performed to identify enriched signaling patterns within each signature (“Gene set enrichment analysis on RNA-Seq signatures” in Additional file 1: Supplementary results; Additional file 1: Tables S3–S9; Additional file 3).

**Fig. 2:**
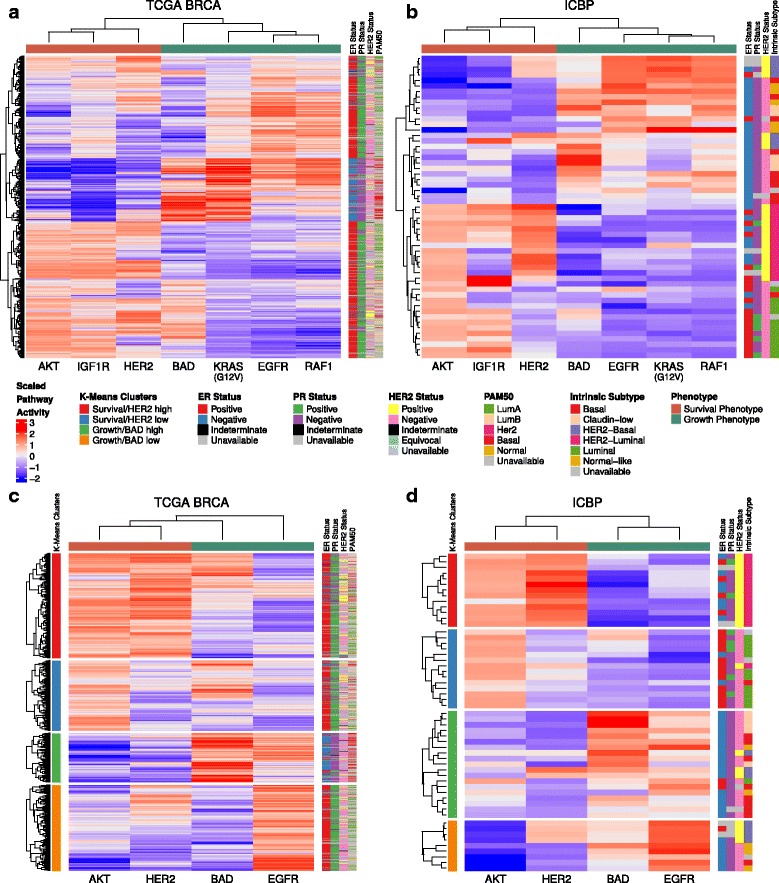
Analysis of pathway activity and intrinsic subtypes in **a** 1119 TCGA breast cancer samples and **b** 55 ICBP breast cancer cell lines. HER2, IGF1R, and AKT and BAD, EGFR, KRAS (G12V), and RAF1 pathway activities reveal two distinct clusters that were negatively associated. GFRN characterization reveals a dichotomy in TCGA breast cancer patients, high BAD/EGFR/KRAS/RAF1 (growth phenotype; column color label shown in *aquamarine*) and high HER2/IGF1R/AKT (survival phenotype; column color label shown in *coral*). Subtypes determined by immunohistochemistry and intrinsic subtyping are shown on the *right side row color labels*. **c** K-means clustering of TCGA samples identifies subsets of samples within the survival phenotype that have high HER2 activation and low HER2 activation, and subsets of samples within the growth phenotype that have high BAD activation and low BAD activation (shown in the *left side row color labels*). **d** These clusters are also seen in ICBP

Finally, unsupervised hierarchical clustering of the pathway activity estimates for all GFRN signatures in both ICBP cell lines and TCGA patient data resulted in a dichotomous pattern (Fig. 2a, b). The HER2, IGF1R, and AKT pathways formed a cluster, as did the remaining BAD, EGFR, KRAS, and RAF1 pathways (Fig. 2a, b). There was some overlap between the two clusters, likely due to the known crosstalk and compensation that occurs between the PI3K and MAPK pathways [81]. In general, however, when one set of pathways was high, the other set was low, which shows that samples expressed a dominant phenotype of GFRN activity. These results strongly suggest a pathway-level dichotomization of the GFRN, which is represented by two primary phenotypes: (1) activation of the HER2/IGF1R/AKT pathways or “survival phenotype”; (2) activation of the BAD/EGFR/KRAS/RAF1 pathways or “growth phenotype.”

After identifying the two main dichotomous GFRN phenotypes, these phenotypes were investigated for how they related to classic IHC-based subtypes, intrinsic subtypes, and additional heterogeneity present within each phenotype (Fig. 2). To investigate if these phenotypes were independent of ER status, pathway activity estimates were clustered for ER+ and ER− samples separately for both ICBP and TCGA samples. The pathway activity bifurcation pattern, as represented by GFRN phenotypes, was consistent within ER+ and ER− samples, indicating GFRN phenotypes are partially independent of ER status (Additional file 1: Figure S4). The variability between histological and intrinsic subtypes can also been seen in the heatmap sidebars for TCGA and ICBP data (Fig. 2a–d), and in boxplots of pathway activity estimates across clinical and intrinsic subtypes in TCGA (Additional file 1: Figures S5 and S6). Samples classified as the survival phenotype included samples from all histological and intrinsic subtypes (Additional file 1: Tables S10 and S11 and Figure S7). Of the 596 TCGA tumors from the survival phenotype, 84.74% were ER+, 72.99% were PR+, 18.12% were HER2+, and 26.51%, 17.79%, 6.88%, and 0.34% were of luminal A, luminal B, HER2-enriched, and basal subtypes, respectively. For the growth phenotype (n = 523), even more heterogeneity in ER, PR, and HER2 status was observed (ER+, 53.54%; ER−, 37.67%; PR+, 46.85%; PR−, 43.98%; HER2+, 10.33%; HER2−, 56.41%; basal, 17.78%; Her2 enriched, 3.06%; luminal A, 13.96%; and luminal B, 4.02%). Hence, clinical and intrinsic subtypes varied in each phenotype cluster, and the GFRN phenotypes provide additional information which complements existing breast cancer clinical and intrinsic subtypes in both patient and cell line data [14, 37, 82, 83].

HER2 activity differences were also observed within the survival phenotype, and differences in BAD activity within the growth phenotype. To further classify samples specifically on these differences, k-means clustering was performed on the AKT, BAD, EGFR, and HER2 pathway activity predictions in ICBP and TCGA. The four resulting clusters separated the survival phenotype into two subsets of samples that had either high or low HER2 activity, and the growth phenotype into two subsets of samples that had either high or low BAD activity. These patterns were observed in both TCGA and ICBP datasets (Fig. 2c, d). Again, subtype plotted against these four subgroups as presented in the sidebars reveal there is additional heterogeneity within ER and PR status that is captured using GFRN subgroups. Of note, a survival analysis of the four subgroups in TCGA did not show significant differences in survival (λ^2^ = 5.5, *p* value = 0.141; Additional file 1: Figure S8). This indicates that these subgroups may not relate to survival directly. Instead, these subgroups discriminate aberrant pathway activity that may help select patient subgroups likely to respond to specific drugs targeting those pathways. GFRN phenotypes complement ER status and current subtyping methods, but are more biologically focused than current intrinsic subtypes and are useful in addition to current IHC-based subtypes.

### GFRN phenotypes and subgroups contribute to variation found in TCGA breast cancer gene expression data

In order to determine if the GFRN phenotypes and subgroups contributed to heterogeneity in the breast cancer data using an unbiased approach, an unsupervised PCA was performed on 1119 breast cancer RNA-Seq samples from TCGA. PCA is a dimension reduction method capable of identifying uncorrelated sources of variation within a dataset as principal components (PCs) [84, 85]. The first five PCs identified in this dataset represented the most significant amount of variability explaining 34.3% of the total variance. The remaining components, each accounting for less than 4% of the total variation, were not investigated due to their minor contribution to total variance. Of note, PC 1 was significantly associated with average gene expression of the samples (Spearman’s correlation −0.786, *p* value <0.0001), potentially reflecting technical and non-disease-related sample variation (Additional file 1: Figure S9). However, PC 1 was included in analyses to demonstrate its performance. To explain variability as presented by PC values, currently used histological (ER, PR, and HER2) and intrinsic subtypes were compared to GFRN-based approaches. First, each classification approach was investigated if it explained variability in each PC. When comparing PC values, significant differences were found between ER+ and ER− samples and PR+ and PR− samples for PCs 1 through 5, between HER2+ and HER2− samples for PCs 3, 4, and 5, across intrinsic subtypes for PCs 1 through 5 (ANOVA, *p* value <0.0001), between growth and survival phenotypes for PCs 2 through 5, and across four GFRN subgroups for PCs 1 through 5 (ANOVA *p* value <0.0001). These results indicate that significant variation underlying TCGA breast cancer data may be contributed from multiple sources, including GFRN phenotypes, subgroups, and histological and intrinsic subtypes.

Second, a linear modeling approach was used to model the first five PCs with GFRN subgroups, intrinsic subtypes (PAM50), and histological (ER, PR, and HER2) subtypes. Variance explained by each model was compared in terms of R^2^ values. We included 355 TCGA tumor samples for which all of these variables were available. ER (R^2^ = 0.56) and PR (R^2^ = 0.407) status explained a significant proportion of PC2 but explained less than 10% of the total variability in the other PCs. HER2 status alone explained less than 4% of the variability for any of the PCs. Both GFRN subgroups, and intrinsic subtypes, explained additional variability in PCs 1–5. For all five PCs, adding the GFRN subgroups or intrinsic subtypes to clinical subtypes increased the R^2^ values of the model (*p* value <0.01 for all models tested; Additional file 1: Figure S10 and Table S12). Specifically, adding GFRN subtypes to a model of PCs explained an additional 10–35% (*p* value <0.00001) of the variation when compared to a model of ER status alone while PAM50 explained only 4–20% of the variation (Additional file 1: Table S12).

On a more granular level, GFRN subgroups explained an additional 13.5% (*p* value <0.00001) of the variability for PC2, which was not explained by ER status alone. For PC3, GFRN subtypes explained an additional 35% of the variation when compared to a model of ER status alone (ER R^2^, 0.052; ER+ GFRN subtype R^2^, 0.398; *p* value <0.00001) and intrinsic subtypes only explained an additional 20% of the variation compared to the same model of ER status alone (ER+ intrinsic subtype R^2^, 0.254; *p* value <0.00001). Overall, the models that contained GFRN subgroups explained a larger percentage of the variance of PC 1, 3, and 4, and models that contained intrinsic subgroups explained a larger percentage of the variance of PCs 2 and 5 (Additional file 1: Figure S10). These significant R^2^ and *p* values confirm the non-redundancy of GFRN subgroups in relation to commonly used clinical features in breast cancer. Additionally, GFRN subgroups explain additional variance in models of PCs 1, 3, and 4 compared to models containing intrinsic subgroups.

**Fig. 3:**
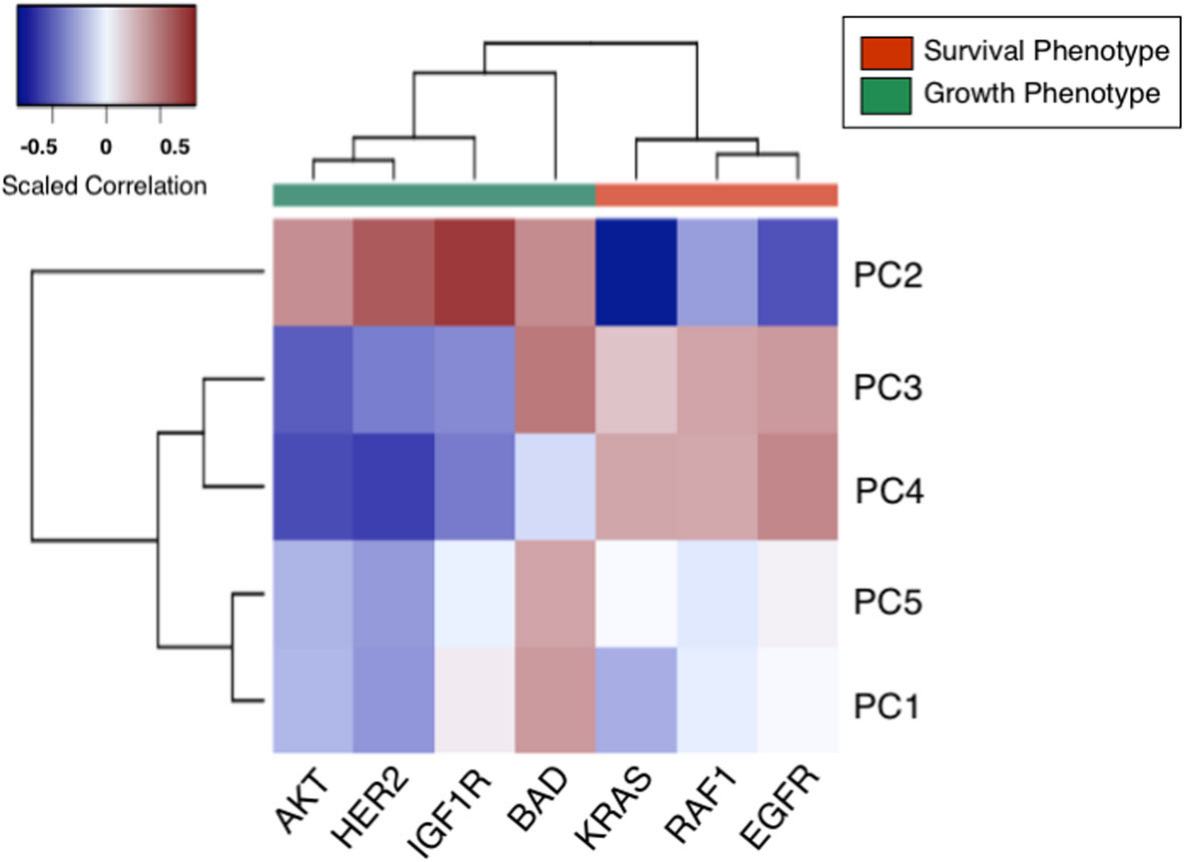
Principal component analysis across TCGA breast tumors. Correlation heatmap between principal component (PC) values from PCs 1 through 5 and ASSIGN GFRN pathway estimates from TCGA breast cancer RNA-Seq data. *Red colors* represent a positive correlation and *blue colors* represent a negative correlation

Next, the variability contributed by GFRN subgroups was investigated in relation to biological signals, or pathway activity in this case. PC values for PCs 1 through 5 were correlated with the GFRN pathway activation estimates from TCGA (Fig. 3; Additional file 1: Table S13). Again, a striking bifurcated pattern was found in the correlations between pathway activity and PCs in this independent variability analysis. PC 2 was positively correlated with EGFR, KRAS, RAF1, and BAD activation and negatively correlated with HER2, IGF1R, and AKT activation. Therefore, PC 2 is demonstrating characters of the growth phenotype. PCs 3 and 4 were positively correlated with HER2, IGF1R, and AKT activation and negatively correlated with EGFR, KRAS, RAF1, and BAD activation, thus representing growth phenotype characteristics (Fig. 3). Both PC 1 and PC5 were negatively correlated with EGFR and RAF1 activation but positively correlated with BAD activation. Since intrinsic subtypes are derived empirically without pointing to any specific biological phenomenon, a correlation to intrinsic subtypes could not be performed.

In summary, these novel GFRN subgroups explained a significant amount of variability in TCGA RNA-Seq data. The GFRN subgroups described variation beyond ER, PR, and HER2 status in all cases, and beyond intrinsic subtypes for three out of five cases. These results suggest that variability in breast cancer data can be further explained in terms of the GFRN pathway activity. Therefore, GFRN subgroups can augment current breast cancer subtyping methods by encompassing additional heterogeneity not captured by traditional approaches. This pathway-based approach may further explain specific variation in terms of pathway activity, which may point to identifying therapeutic targets.

### Breast cancer growth phenotypes bifurcate in expression of mitochondrial apoptotic proteins

Next, differences between the survival and growth phenotypes were examined at the biological level, specifically in terms of mitochondrial-mediated intrinsic apoptosis mechanisms. Although cytotoxic anticancer agents induce cell death through various mechanisms, including intrinsic or extrinsic apoptosis, necrosis, autophagy, or mitotic catastrophe [86, 87], we focused on mitochondrial-mediated intrinsic apoptosis mediated by BCL-2 family proteins for the following reasons. First, BCL-2 family members, which regulate the commitment to mitochondrial apoptosis by balancing pro-apoptotic proteins such as BAD and BIM, and anti-apoptotic proteins such as BCL-2 or MCL-1 [20], have been shown to contribute to the formation, progression, and therapeutic response in breast and other cancers [21, 88]. Second, particular GFRN signaling pathways, such as those found in the survival and growth phenotypes, have the potential to induce apoptosis resistance by dysregulating BCL-2 family proteins, suggesting that targeting GFRN members may lead to increased apoptosis [23–29, 89–91]. Third, several therapeutic strategies targeting anti-apoptotic BCL-2 family members are currently under investigation; therefore, understanding which BCL-2 proteins each phenotype is expressing may provide insight into additional treatment strategies for breast cancer [22, 92–94].

**Fig. 4:**
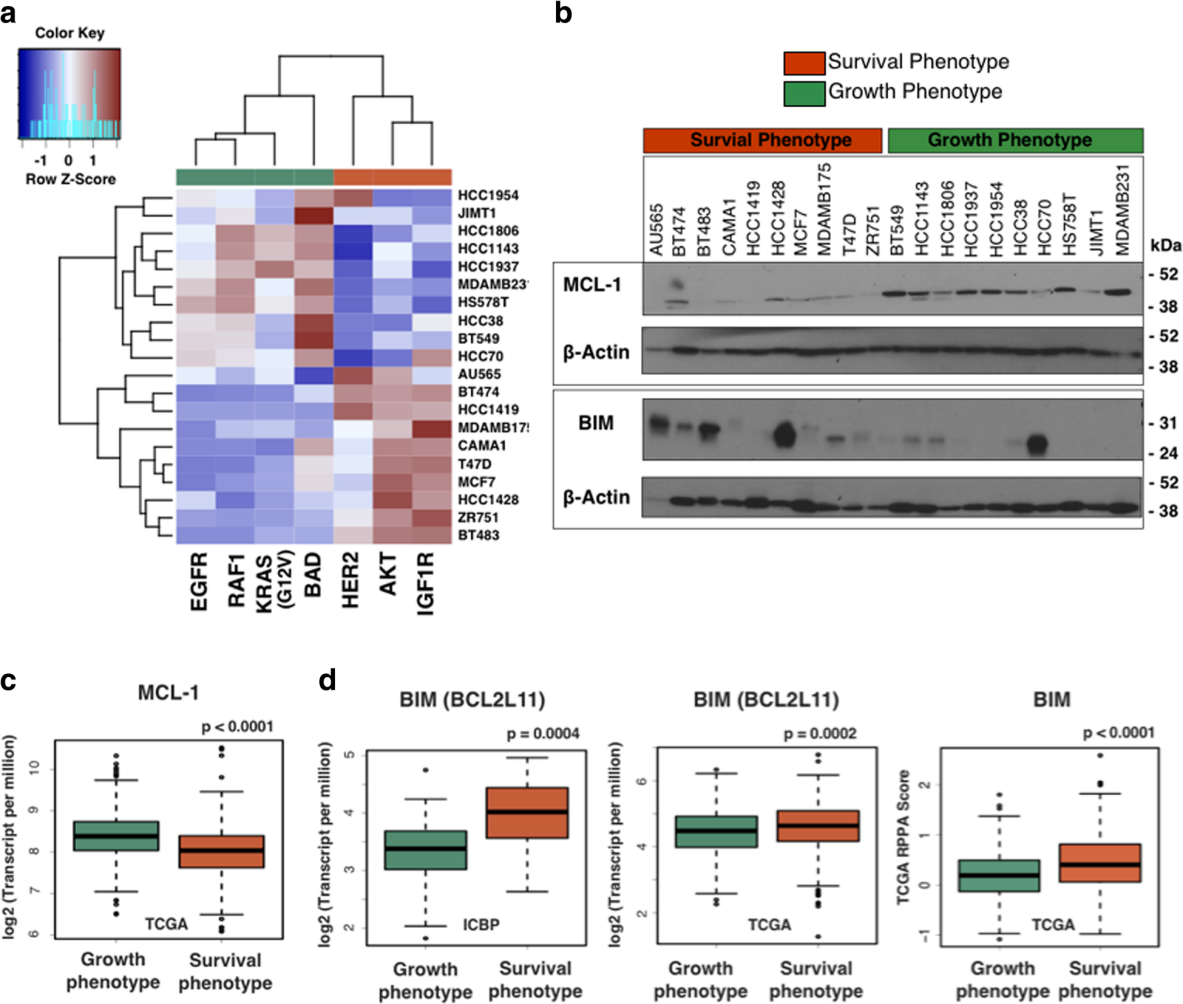
Survival and growth phenotypes differ in cell survival mechanisms. **a** The heatmap represents scaled activation values across 20 breast cancer cell lines used in this analysis for each GFRN pathway. **b** Western blot analysis for MCL-1, BIM, and B-actin control across 20 breast cancer cell lines of either the survival phenotype or growth phenotype. **c, d** Boxplots between samples classified as the survival phenotype or growth phenotype for **c** MCL-1 gene expression (log2 (Transcript per million)) in TCGA data, **d** BIM gene expression (log2 (Transcript per million)) in TCGA and ICBP data, and protein expression (RPPA score) in TCGA data. Student *t*-tests were performed to determine significance

Here, Western blotting was used to investigate whether protein expression of particular BCL-2 family members differed in breast cancer cell lines classified as the survival or growth phenotypes (Fig. 4). The pro-apoptotic protein BIM and anti-apoptotic protein MCL-1 were probed across ten breast cancer cell lines of the survival phenotype (eight ER+, two ER−), and ten cell lines of the growth phenotype (ten ER−) (see Additional file 2 for cell line characteristics). Higher levels of MCL-1 were found in cell lines of the growth phenotype, and higher levels of BIM were found in the survival phenotype (Fig. 4b). To determine if differences in MCL-1 and BIM protein expression between the survival and growth phenotypes were due to other properties, such as ER status, a Western blot assay was performed using cell lines with additional heterogeneity in ER status. Although limited by the number of ER+ cell lines of the growth phenotype, 12 cell lines belonging to the survival phenotype (five novel ER+, three ER+ repeats from previous assay, and four novel ER−) and seven cell lines from the growth phenotype (one novel ER+, two novel ER−, and four ER− repeats) were included. The protein expression of MCL-1 and BIM were not strictly dependent on the ER status (Additional file 1: Figure S11).

To understand if similar results could be found in patient tumors, the expression of BCL-2 family member genes was examined, and *MCL-1* gene expression was found to be higher in the growth phenotype of TCGA patient tumors (n = 523) versus the survival phenotype (n = 596, *p* < 0.0001) (Fig. 4c). These results were consistent with previous studies showing that EGFR signaling can upregulate gene expression of *MCL-1* [25, 89–91]. In addition to MCL-1 dysregulation, breast cancer cell lines of the growth phenotype expressed lower levels of the pro-apoptotic protein BIM (Fig. 4d). In support of this assessment, lower levels of BIM (*BCL2L11*) gene expression were found in ICBP breast cancer cell lines (*p* = 0.0004) and TCGA tumors (*p* = 0.0002), and RPPA protein expression was lower in TCGA tumors (*p* < 0.0001) (Fig. 4d). These results concur with literature showing that EGFR signaling through ERK activation can lead to repression of BIM [27–29]. Also, the co-occurrence of high MCL-1 levels and low BIM levels in the growth phenotype are likely due to MCL-1’s known ability to bind and neutralize BIM, which leads to prevention of apoptosis death effector activation [21, 95]. In summary, these results show an interesting mitochondrial apoptotic pathway induction that is dependent on GFRN activity. Specifically, breast tumors classified as the growth phenotype may overexpress MCL-1 and inhibit BIM expression to achieve cell survival. These findings illustrate that breast cancer phenotypes, defined by activation of specific growth factor receptor pathways, express different apoptotic proteins and may resist apoptosis differently.

### GFRNs predict drug response in breast cancer

**Fig. 5:**
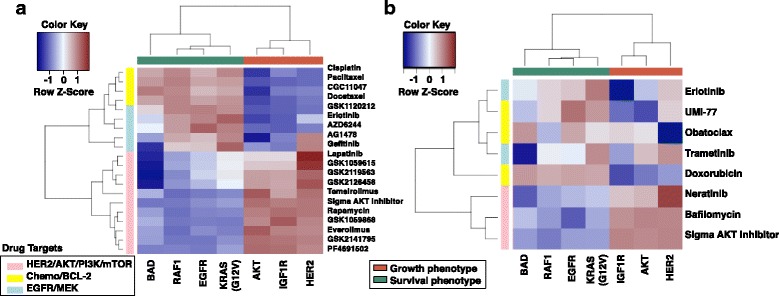
Growth factor receptor network phenotypes reflect dichotomous drug response in breast cancer cell lines. *Colors* correspond to scaled Spearman correlations between specific pathway activation estimates generated with ASSIGN and drug sensitivity (−logGI50) across **a** 55 breast cancer cell lines from the ICBP panel and **b** 23 breast cancer cell lines in an independent drug assay. *Red* represents positive correlation and *blue* represents negative correlation. Pathways cluster across the *x-axis* as AKT growth phenotype (*coral color*) and EGFR growth phenotype (*green*). Drug classes are represented along the *y-axis*: pink, HER2/AKT/PI3K/mTOR-targeted therapies; yellow, chemotherapies/BCL-2 targeting therapies; and blue, EGFR/MEK-targeted therapies

Since there was a clear dichotomy in the GFRN signaling mechanisms between the survival and growth phenotypes, these phenotypes were investigated in relation to drug response in breast cancer cell lines. Pathway activation estimates were correlated with drug response data for 90 drugs from the ICBP breast cancer cell line panel. Importantly, a consistent bifurcation pattern was observed for drug response in the cell line data that matched the observed pathway-level bifurcation. Specifically, cancer cells classified as expressing the survival phenotype were sensitive to therapies that target AKT, PI3K, HER2, and mTOR (Fig. 5a). Additionally, these cell lines were more resistant to chemotherapies and targeted therapies that block EGFR and MEK. In contrast, cancer cells expressing the growth phenotype were sensitive to chemotherapeutics such as docetaxel, paclitaxel, and cisplatin. These cell lines were also sensitive to EGFR- and MEK-targeted therapies, but more resistant to AKT, PI3K, HER2, and mTOR inhibitors (Fig. 5a).

This dichotomy in drug response of the survival and growth phenotypes was further tested in an independent drug response assay. Eight drugs on a panel of 23 breast cancer cell lines were tested (see Additional file 2: Sheet 1 for cell lines), and cell viability was tested upon drug treatment by measuring ATP levels. Drugs included were obatoclax (BCL-2, BCL-XL, BCL-W, BAK inhibitor), UMI-77 (selective MCL-1 inhibitor), erlotinib (EGFR inhibitor), doxorubicin (topoisomerase II inhibitor), trametinib (MEK inhibitor), neratinib (pan-HER tyrosine kinase inhibitor), Sigma-Aldrich AKT1/2 inhibitor (dual AKT1/2 inhibitor), and bafilomycin (apoptosis inducer that inhibits PI3K/AKT signaling and autophagy inhibitor) at different doses (Additional file 2: Sheet 2). Again, a discrete pattern was observed between the survival and growth phenotypes that translated to a bifurcated drug response pattern (Fig. 5b). Responses to the chemotherapy (doxorubicin) and the EGFR pathway inhibitor (erlotinib) were high for the growth phenotype. In contrast, cancer cell lines classified as the survival phenotype responded well to drugs targeting components of the PI3K pathway, such as Sigma-Aldrich AKT1/2 inhibitor, neratinib, and bafilomycin.

In addition to the bifurcation of GFRN and drug response, breast tumor cells of the growth phenotype showed a higher response to the specific MCL-1 inhibitor UMI-77 (Fig. 5b). This is consistent with the findings that samples within the growth phenotype have higher MCL-1 expression than the survival phenotype. Response to obatoclax could not be clearly distinguished based on these phenotypes, likely due to its nonspecific binding to pro-survival proteins, including BCL-2, BCL-XL, and MCL-1 [96]. Overall, the GFRN phenotype-based drug response predictions were validated in this independent drug response assay. Additionally, drug sensitivity of emerging therapies such as UMI-77, neratinib, and bafilomycin showed differences between the two phenotypes, further highlighting the close relationship between GFRN signaling activity and response to therapies directed at pathways in this network.

**Fig. 6:**
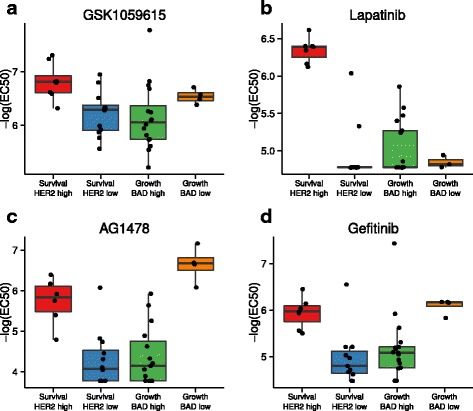
Differential drug response identified in GFRN phenotype heterogeneity. Boxplots of –log (EC50) drug response data from four drugs in the drug assay that show a differential drug response within growth factor phenotypes. **a** GSK1059615, a PI3K and mTOR inhibitor, caused an increase in response in samples within the survival phenotype classified as having high HER2 activity. **b** Lapatinib, a HER2 inhibitor, stimulated a stronger response in samples within the survival phenotype with high HER2 activity. **c** AG1478 and **d** gefitinib, EGFR inhibitors, caused an increased response in samples within the growth phenotype classified as having low BAD activity

When GFRN phenotype subgroups were considered, several drugs in the ICBP drug response assay showed significantly different drug response profiles in the subgroups found in each GFRN phenotypic arm. For example, the PI3K and mTOR inhibitor GSK1059615 and HER2/EGFR-targeting drug lapatinib were more effective in cell lines within the survival phenotype showing higher HER2 activity (*p* = 0.009 and *p* < 0.000001, respectively; Fig. 6a, b). Additionally, ICBP cell lines expressing the growth phenotype responded better to EGFR-targeting drugs AG1478 and gefitinib in the EGFR/BAD low cluster compared to the EGFR/BAD high cluster (*p* = 0.001 and *p* = 0.001, respectively; Fig. 6c, d).

To determine if this bifurcation pattern was independent of clinical and intrinsic subtyping approaches, the correlations between pathway activation and drug response for ER+ and ER− and HER+ and HER− ICBP cell lines were clustered separately. Again, cell lines with high AKT/IGF1R/HER activity, i.e., the survival phenotype, were more sensitive to HER2/AKT/PI3K-targeted drugs even within ER− and HER− cell lines (Additional file 1: Figure S12). In ER+ and HER+ cell lines, many PI3K/AKT/HER2-targeting drugs are more effective in the survival phenotype, as expected. However, there was additional drug response heterogeneity within ER+ samples that is associated with variations in BAD and HER2 pathway activity. These subgroups are thus helpful to further classify samples for better drug response prediction. To assess drug response across ER, PR, and HER2 status and intrinsic subtypes, it was found that out of 90 drugs studied in ICBP only 13 (14.4%), 12 (13.3%), and 19 (21.1%) showed significant differences in drug response based on ER, PR, and HER2 status, respectively, but growth/survival phenotypes were significant for 27 (49%) (Additional file 1: Table S14). As further evidence, while HER2 positive status is a biomarker for effective HER2-targeted therapy, drug sensitivity does not solely depend on HER2 status. For example, while HER2 status performs much better in differentiating lapatinib’s response than ER and PR status (*p* < 0.0001), some HER2− cell lines, such as HCC70 and 184A1, may respond to lapatinib (Additional file 1: Figure S13a–c). The subgroup analysis showed the survival/HER2 high subgroup to be more sensitive to lapatinib than any other subgroup (Fig. 6b). In contrast, intrinsic subgroup analysis showed, in general, that the luminal subtype was more sensitive, but significant variability in lapatinib sensitivity exists within the luminal subtype (Additional file 1: Figure S13d). Other detailed examples describing comparisons between the GFRN phenotypes and other methods are included in Fig. 6. In conclusion, the GFRN phenotypes provide additional information to current approaches; GFRN phenotypes and subgroups could be used to further stratify samples and may help select more appropriate candidates for effective drug response.

## Discussion

**Fig. 7:**
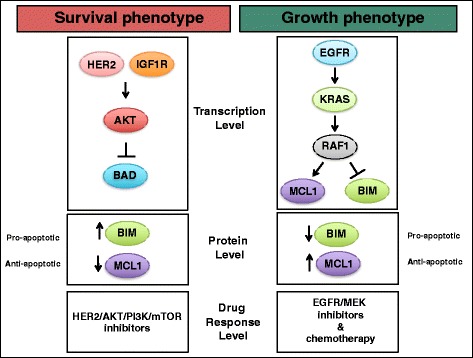
Summary of the survival and growth phenotypes in breast cancer. The survival phenotype is characterized by high HER2, IGF1R, and AKT pathway activation, high expression of pro-apoptotic BIM, low expression of anti-apoptotic MCL-1, and response to HER2, AKT, PI3K, and mTOR inhibitors. The growth phenotype is characterized by high EGFR, KRAS, and RAF1 activation, high expression of MCL-1, low expression of BIM, and response to EGFR/MEK-targeted therapies and chemotherapies

Targeted therapies directed against the key members of the growth factor receptor network (GFRN), such as EGFR, PI3K, AKT, and mTOR inhibitors, are currently in preclinical development, clinical trials, or approved for use in breast cancer [16]. However, predicting patients’ responses to therapies is challenging due to difficulties in measuring complex signaling events in tumors. Here, this issue was addressed by investigating global GFRN activity in breast cancer using these novel signatures. Two discrete patterns of GFRN pathway activity, or phenotypes, were found (Fig. 7). The survival phenotype was characterized by the activation of the HER2, AKT, and IGF1R pathways, and the growth phenotype by the activation of the EGFR, KRAS, RAF1, and BAD pathways. Additional subgroups were also found within the survival and growth phenotypes, including HER2 high and low activity groups within the survival phenotype and BAD high and low activity groups within the growth phenotype. Although these discrete phenotypes were named the survival and growth phenotypes for simplicity, GFRN pathways comprising both groups can contribute to growth and survival. To the best of our knowledge, this is the first study to characterize GFRN activity using signature-based representations of activity across multiple pathways.

These discrete subgroups displayed differences in response to targeted therapies and chemotherapies in breast cancer cell lines. For example, conventional chemotherapies such as docetaxel, paclitaxel, and doxorubicin were more effective for the growth phenotype than the survival phenotype. Sensitivity to PI3K, HER2, AKT, and mTOR inhibitors and resistance to conventional chemotherapies were also found in the survival phenotype. Among the subgroups, the survival phenotype/high HER2 subgroup was hypersensitive to lapatinib, a HER2 and EGFR dual inhibitor. Similarly, the survival phenotype/high HER2 subgroup was more sensitive to GSK1059615, a PI3K/mTOR inhibitor than the survival phenotype/low HER2 subgroup. Cell lines of the growth phenotype responded better to EGFR and MEK inhibitors and to conventional chemotherapies. The growth phenotype/low BAD subtype was more sensitive to both AG1478 and gefitinib (EGFR inhibitors) than the growth phenotype/high BAD subtype. Overall, the GFRN pathway-based phenotyping contributed to information related to drug response.

Analysis of these novel phenotypes in breast cancer cell lines and tumors also revealed interesting differences in intrinsic apoptosis. For example, breast cancer cell lines and tumors of the growth phenotype had higher levels of the anti-apoptotic protein MCL-1 and lower levels of the critical pro-apoptotic protein BIM. These results are consistent with the notion that the MAPK pathway can activate MCL-1 expression and that activation of ERK1/2 and the MAPK pathway can repress BIM [25, 27–29]. An independent drug assay also showed that the growth phenotypic cell lines responded better to a MCL-1 inhibitor (UMI-77). These results suggest that the patients with growth phenotypic expression may benefit from treatments that increase BIM, i.e., MCL-1 inhibitors, in combination with chemotherapies, EGFR inhibitors, or other inhibitors of the MAPK pathway [97, 98]. Therefore, targeting GFRN members may be an effective therapeutic strategy for inhibiting GFRN pathways and increasing apoptosis [22]. These results highlight that mapping phenotypes, such as growth networks in breast tumors, can be exploited to guide the use of targeted therapies. This study was limited to how GFRN activity related to drug response and cellular intrinsic apoptosis, but it is understood that this is not the sole mechanism by which cancer cells die, and other cell death mechanisms, such as necrosis, autophagy, and mitotic catastrophe, should also be considered. In addition, as the use of cell lines is limited, a larger-scale analysis of apoptotic pathways dysregulation in patient tumor cells of all subtypes will be informative in further detailing how these pathways signal in cancer. These phenotypes many correlate with other subtyping properties, and may also be confounded by properties of intrinsic subtyping.

Importantly, these newly discovered breast cancer survival and growth phenotypes are biologically relevant and offer a direct method for probing and targeting the GFRN in breast tumors. In addition, these phenotypes complement widely used clinical and intrinsic subtypes, and stratification of cancers by these phenotypes leads to enhanced drug response predictions compared to classifying cancers by clinical subtyping approaches. This is most likely because oncogenic pathway activation was measured more comprehensively than relying on single protein measurements. In addition, this approach considers crosstalk between members of the GFRN and correlates with biological processes such as cell survival. This pathway-based approach for identifying phenotypes allows for exploration of additional heterogeneity occurring within the identified phenotypes, which can further improve the ability to stratify breast cancers by pathway activity, which then can be used to predict drug response. Although this method has added to current approaches for predicting drug response in breast cancer, most experiments were performed in breast cancer cell lines with particular classes of drugs; additional drug testing should be performed in breast cancer patient cells in order to confirm these phenotypes.

In summary, a novel genomic pathway-based approach of characterizing the interactive GFRN activation in breast cancer was used to discover two discrete GFRN phenotypes with significant differences in cell survival mechanisms and drug response in breast cancer. These phenotypes captured the distinct bifurcation pattern seen in gene expression, the GFRN pathway activity, mitochondrial apoptotic network protein expression, and drug response (Fig. 7). While ER, PR, HER2 status and, more recently, intrinsic subtype are used to guide breast cancer treatment, these subtyping or classifying approaches may not describe signaling pathway dysregulation in tumor cells. Pathway activity data provide additional information about tumor cells that can be leveraged to predict drug response. Characterizing individual tumors into these phenotypes can help determine which patients will benefit from a treatment and select the appropriate subpopulations for clinical trials. Importantly, these seven pathways did not capture all the heterogeneity of the samples and inclusion of other pathways may have additional benefits. Although feasible, additional investigation is needed before these phenotypes can be used in clinical trials for patient selection, including the testing of these phenotypes in patient primary tumor cells.

## Conclusions

A discriminating bifurcation pattern of key GFRN pathways was identified in breast tumors that expands beyond histological and clinical subtypes. These phenotypes correlated with unique apoptotic and drug response mechanisms. The ability to measure signaling events more accurately in patient tumors advances understanding of the biological basis of cancer. These results may lead to more effective and individualized treatment selection in patients with breast cancer.

## Additional files

Additional file 1: Supplemental results, figures and tables. (PDF 2514 kb)

Additional file 2: The cell lines used in the independent drug assay and the Western blotting experiments, and the drug doses and negative log EC50 values for the independent drug assay. (XLSX 149 kb)

Additional file 3: Full results from the GSVA gene set enrichment analysis for the HER2, IGF1R, AKT, BAD, EGFR, KRAS, and RAF1 signatures. (XLS 1399 kb)

Additional file 4: Optimized gene lists for the AKT, IGF1R, BAD, EGFR, HER2, KRAS, and RAF1 signatures. (XLSX 30 kb)

Additional file 5: Scaled ASSIGN pathway activity predictions, phenotype, and k-means cluster calls for TCGA and ICBP samples. (XLSX 152 kb)
