# Supplementary material for: Activity of distinct growth factor receptor network components in breast tumors uncovers two biologically relevant subtypes
Source: Genome Med. 2017 Apr 26;9:40. doi: 10.1186/s13073-017-0429-x (PMC5406893; doi:10.1186/s13073-017-0429-x)
Supplement: Supplementary file 1 — Supplemental results, figures and tables. (PDF 2514 kb) [file 13073_2017_429_MOESM1_ESM.pdf]

Additional File 1: Supplemental Results, Figures, and Tables

Supplemental Results

Gene set enrichment analysis on RNA-Sequencing signatures. We performed gene set enrichment analysis, using the *Gene Set Variation Analysis for microarray and RNA-seq data* (*GSVA)* method, to better understand the biological significance and discover enriched gene sets between our RNA-sequencing signatures: AKT, BAD, EGFR, HER2, IGF1R, KRAS, and RAF1 and GFP controls. We analyzed 1370 gene sets from the C2: canonical pathways collection from the Molecular Signatures Database (See Methods section in manuscript). Gene sets representing cell cycle pathways were found to be enriched across all signatures, however each signature also showed enrichment for expected and unique gene sets (See Additional File 5.xlxs for full results). For example, the HER2 signature was primarily enriched for immune system and cellular adhesion pathways (Supplemental Table 6; Additional File 1). The IGF1R signature was dominated by metabolic pathways (Supplemental Table 7; Additional File 1). The AKT signature was enriched for immune, apoptotic, and metabolic pathways (Supplemental Table 8; Additional File 1). The BAD signature was enriched for immune system and cell cycle pathways (Supplemental Table 9; Additional File 1). EGFR was dominated by DNA replication and cell cycle pathways (Supplemental Table 10; Additional File 1). KRAS and RAF were highly enriched for MAPK pathways (Supplemental Tables 11- 12; Additional File 1), but RAF also showed enrichment for TGFB and immune system pathways (Supplemental Tables 11-12; Additional File 1). This results highlight the variety of biological pathway differences which can be found by overexpressing GFRN components, further illustrating the need for GFRN pathway activation signatures.

Supplemental Figures

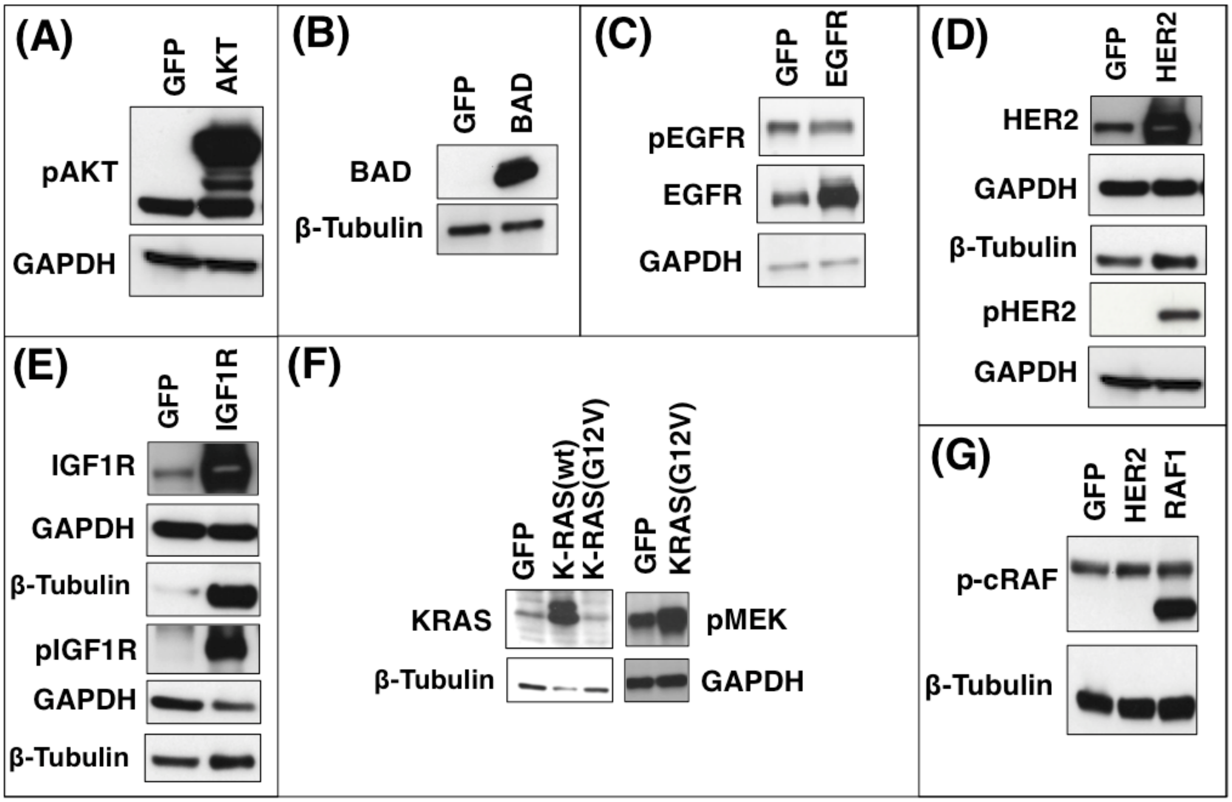

Supplemental Figure 1: Validation of protein overexpression for each GFRN signature. Protein lysates from human primary mammary epithelial cells (HMECs) overexpressing GFRN genes were compared to GFP control protein lysates using Western blotting. **(A)** HMECs overexpressing AKT1 compared to GFP (GAPDH loading control) **(B)** HMECs overexpressing BAD, compared to GFP (β-tubulin loading control) **(C)** HMECs overexpressing EGFR and pEGFR compared to GFP (GAPDH loading control) **(D)** HMECs overexpressing HER2 and pHER2 compared to GFP (GAPDH and β-tubulin loading controls) **(E)** HMECs overexpressing IGF1R and pIGF1R (GAPDH and β-tubulin loading controls) **(F)** HMECs overexpressing pMEK compared to GFP (β-tubulin and GAPDH loading controls) **(G)** HMECs overexpressing RAF1 compared to GFP controls (β-tubulin loading controls).

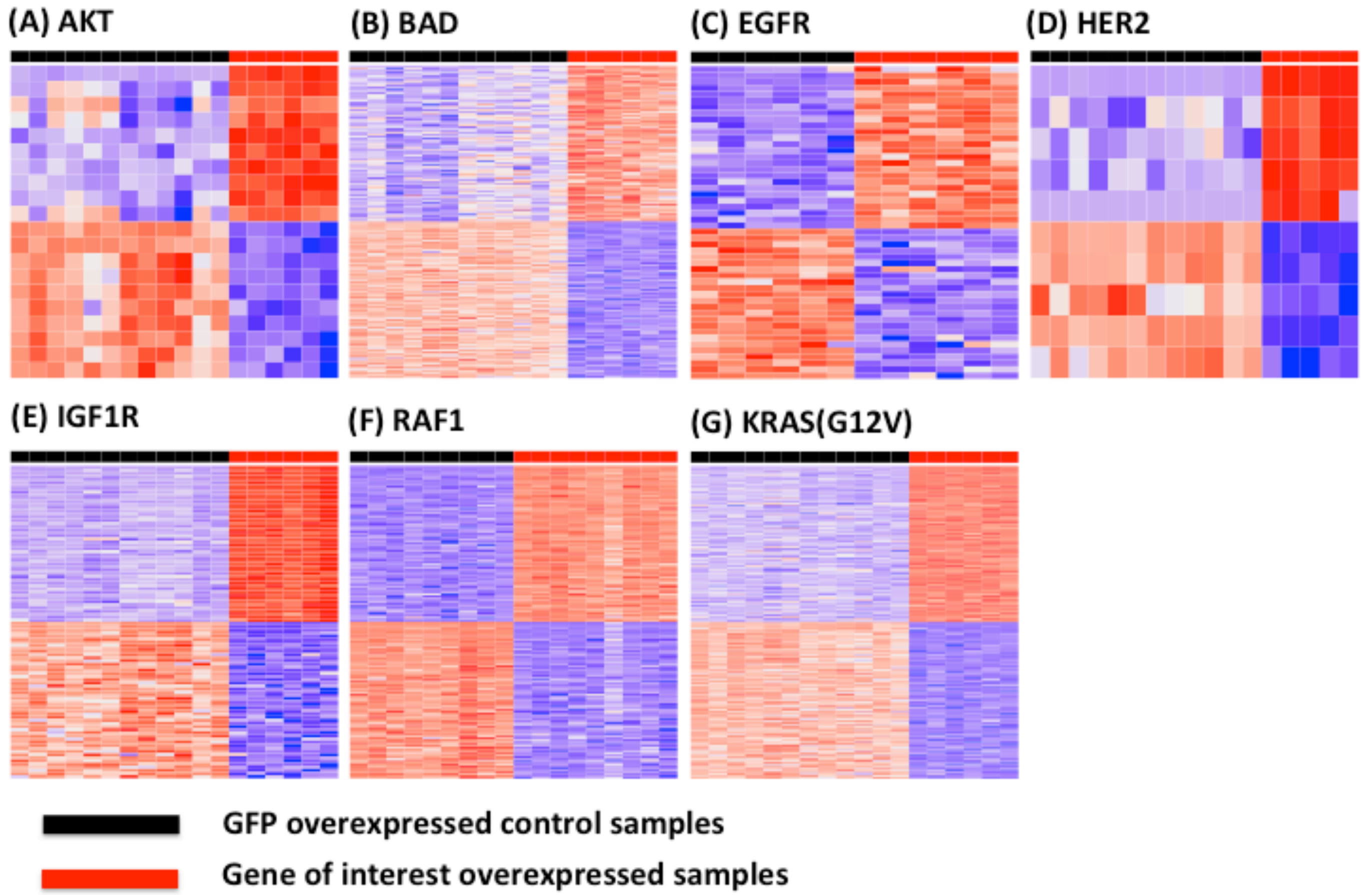

Supplemental Figure 2: Gene expression signatures for key GFRN pathways generated by ASSIGN. (A) AKT 20 gene signature, **(B)** BAD 250 gene signature, **(C)** EGFR 50 gene signature, **(D)** HER2 10 gene signature, **(E)** IGF1R 100 gene signature, **(F)** KRAS (G12V) 200 gene signature, and **(G)** RAF1 350 gene signature. The horizontal black bar indicates green fluorescent protein (GFP) overexpressing control samples, and the red bar indicates the overexpressed genes of interest (i.e., *AKT1, BAD, EGFR, ERBB2 (HER2), IGF1R, KRAS (G12V),* and *RAF1*, respectively) signature samples. The signature genes for each pathway are listed in Additional File 4.

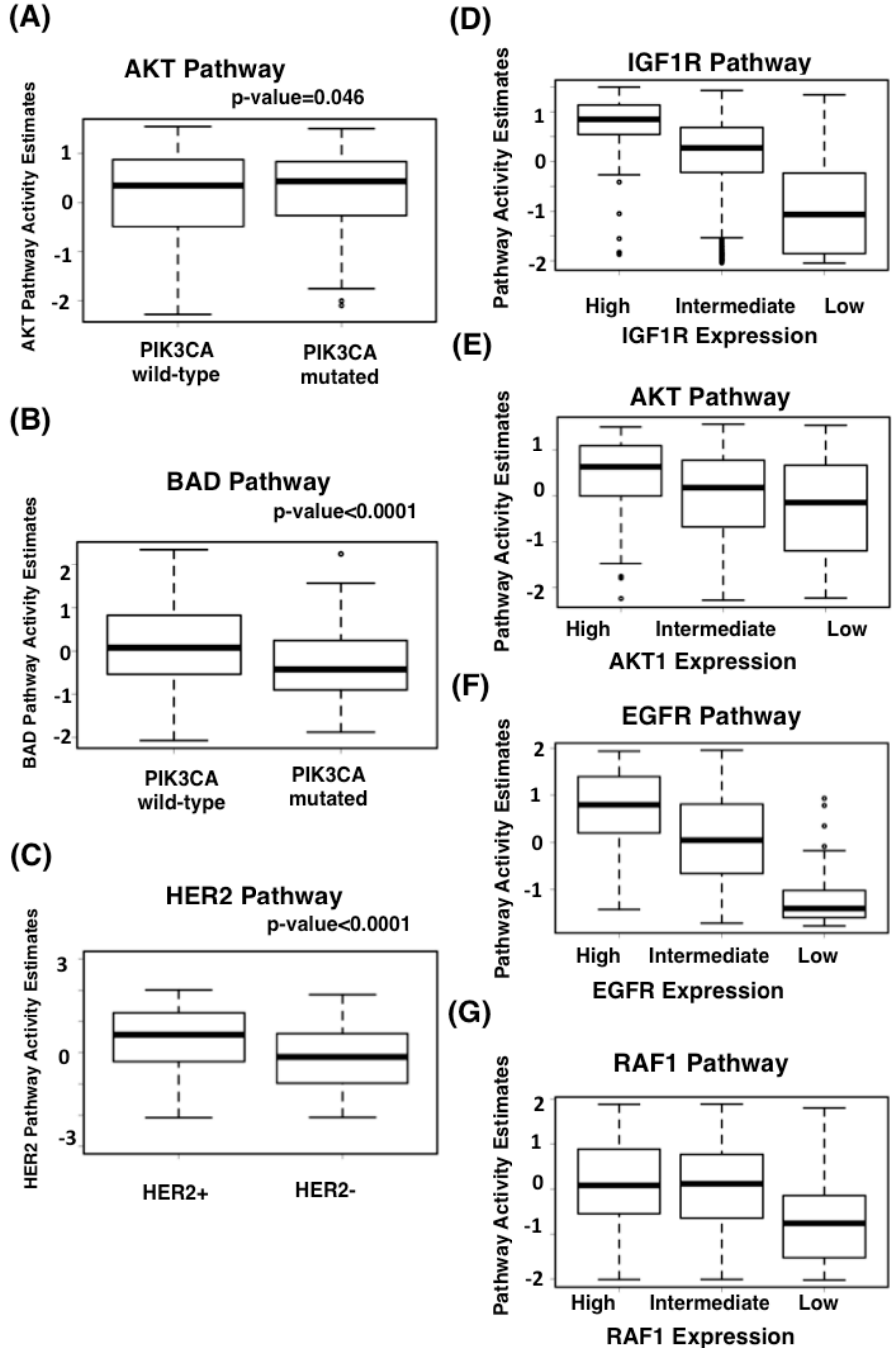

Supplemental Figure 3: Additional GFRN gene expression signature validations in TCGA breast cancer data. Pathway activity estimate boxplots between the **(A)** AKT pathway and **(B)** BAD pathway between *PI3KCA* mutated and *PI3KCA* wild-type TCGA breast cancer samples (n=787). Any mutation in *PI3KC*A was considered pathogenic in this mutation analysis. **(C)** HER2 pathway activation estimates between HER+ and HER- patient TCGA samples (n=708). Pathway activation estimates for **(D)** IGF1R, **(E)** AKT, **(F)** EGFR, and **(G)** RAF1 between 'high', 'intermediate', and 'low' expressing samples in 1119 BRCA TCGA samples. Samples with 90 percentile or higher expression were considered 'high', 10 percentile or lower were considered 'low', and 10 to 90 percentile were considered 'Intermediate' expressing samples for AKT1, EGFR and RAF1. For IGF1R validation, samples with 80 percentile or higher IGF1R expression were considered 'high', 20 percentile or lower was considered 'low', and 20 to 80 percentile expression were considered 'Intermediate' expressing samples.

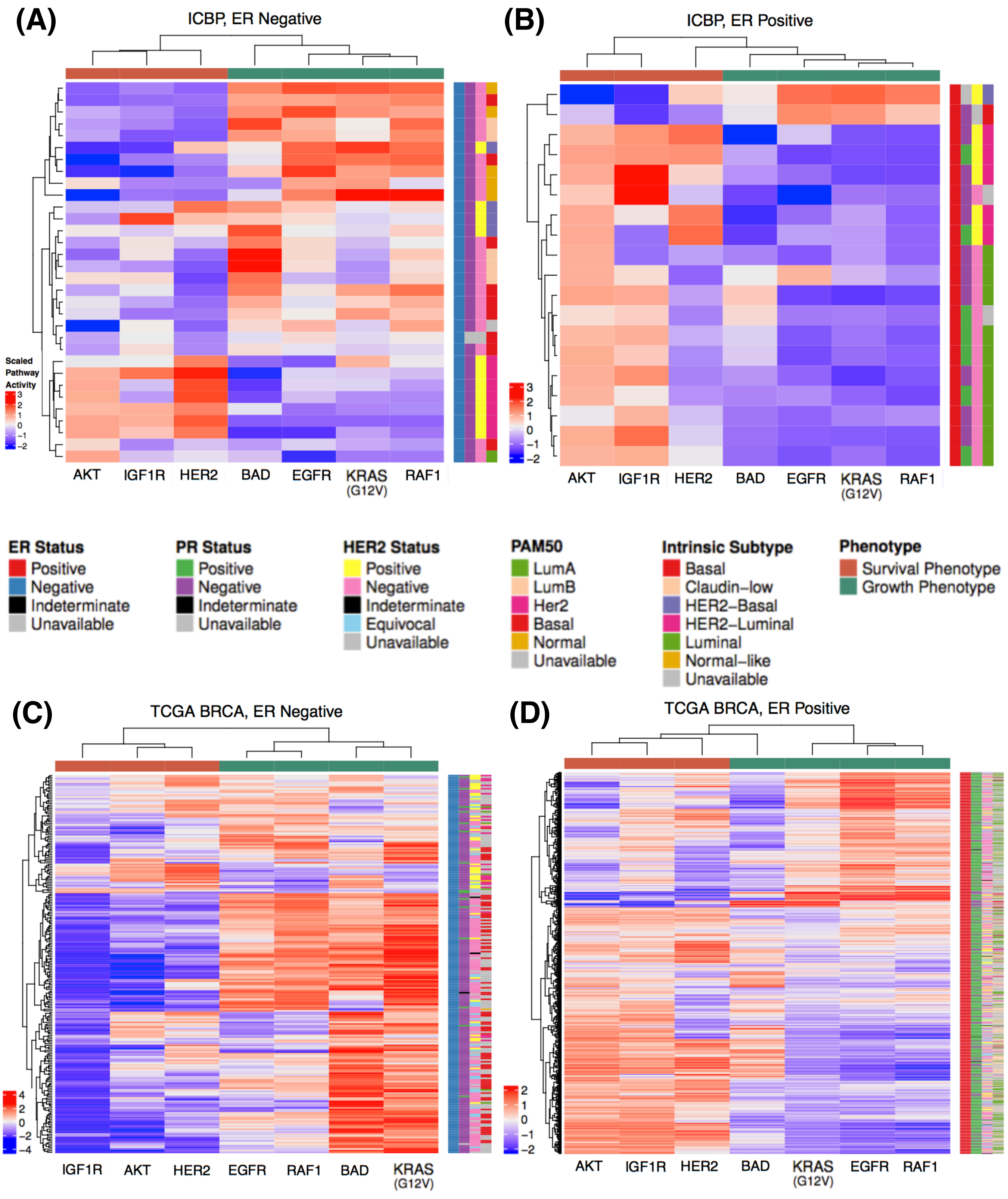

Supplemental Figure 4: Pathway activity estimates between ER+ and ER- samples in breast cancer cell lines and patient data. (A) 19 ER- breast cancer cell lines from ICBP, **(B)** 32 ER+ breast cancer cell lines from ICBP. **(C)** 230 ER- breast cancer patient samples from TCGA, and **(D)** 785 ER+

breast cancer patient samples from TCGA. The growth phenotype is represented in aquamarine above the heat map, and the survival phenotype in coral. Subtypes determined by immunohistochemistry (ER, PR, and HER2), intrinsic subtyping, and PAM50, are label in the right side of the heatmap.

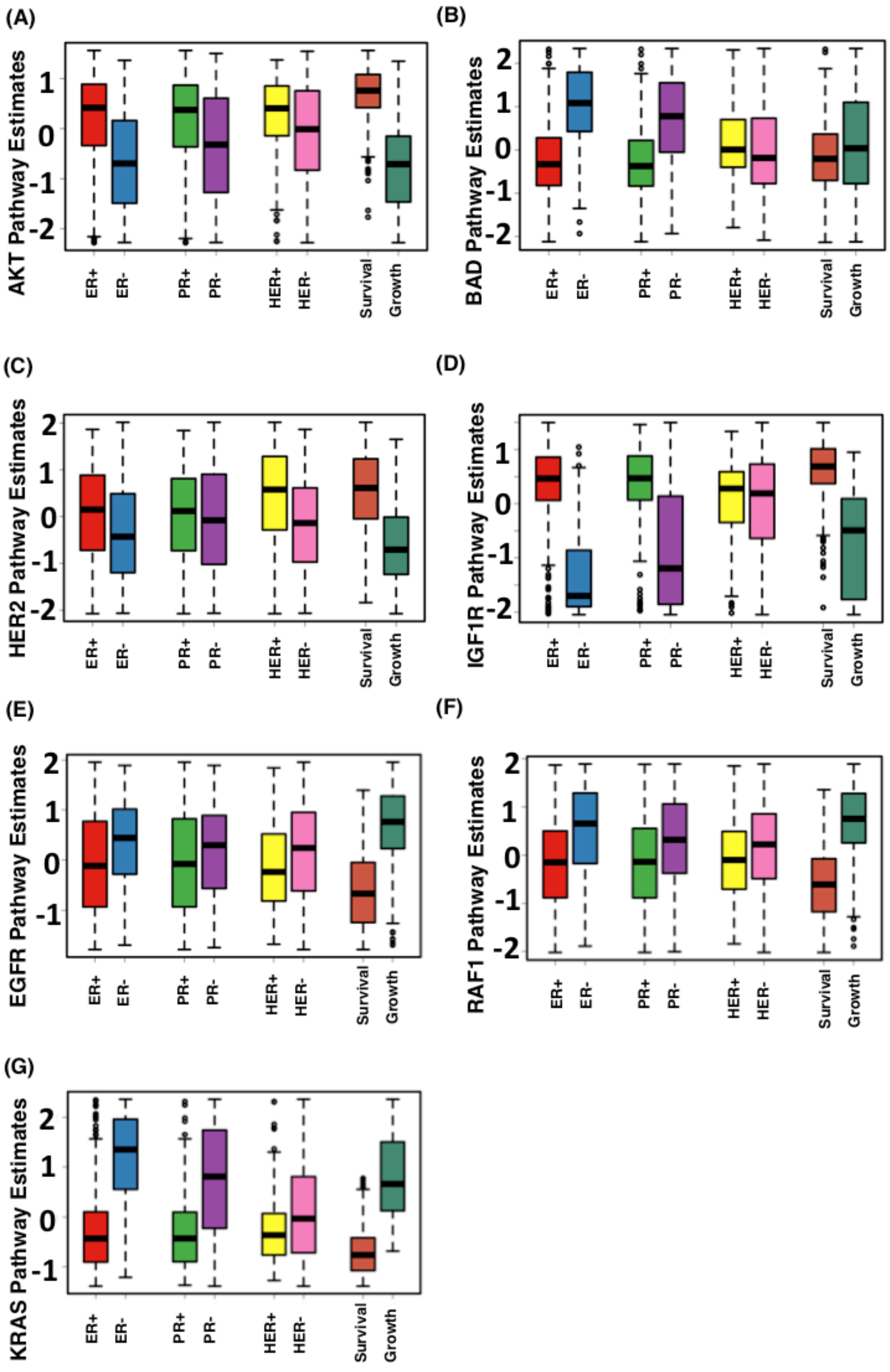

Supplemental Figure 5: Pathway activation estimates across clinical subtypes (IHC-based, N=1012) in TCGA breast cancer data for (A) the AKT pathway **(B)** the BAD pathway **(C)** the HER2 pathway **(D)** the IGF1R pathway **(E)** the EGFR pathway **(F)** the RAF1 pathway **(G)** the KRAS pathway.

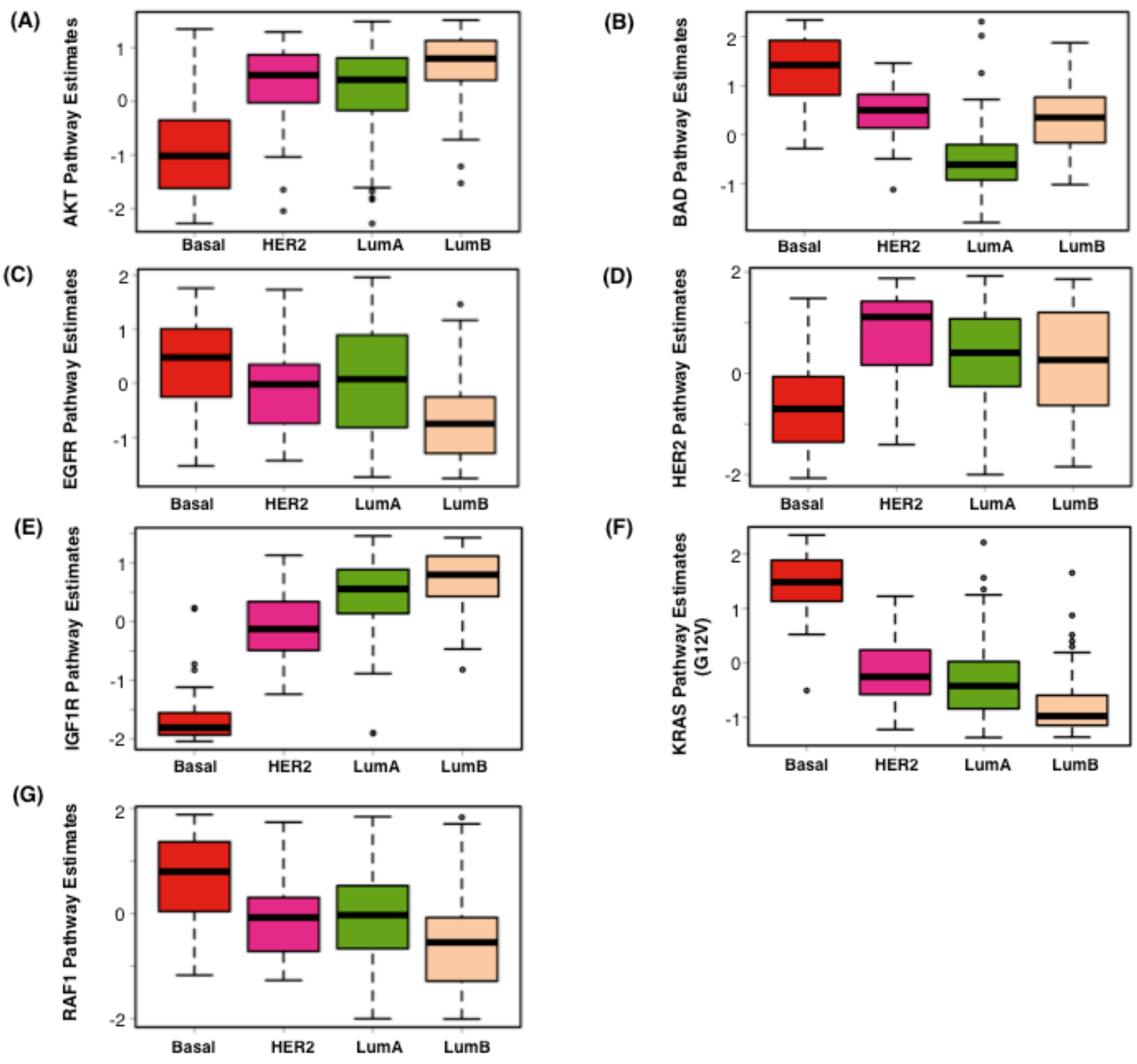

Supplemental Figure 6: Pathway activation estimates across intrinsic subtypes (PAM50 based, N=510) in TCGA breast cancer data for (A) the AKT pathway **(B)** the BAD pathway **(C)** the EGFR pathway **(D)** the HER2 pathway **(E)** the IGF1R pathway **(F)** the KRAS pathway **(G)** the RAF1 pathway estimates.

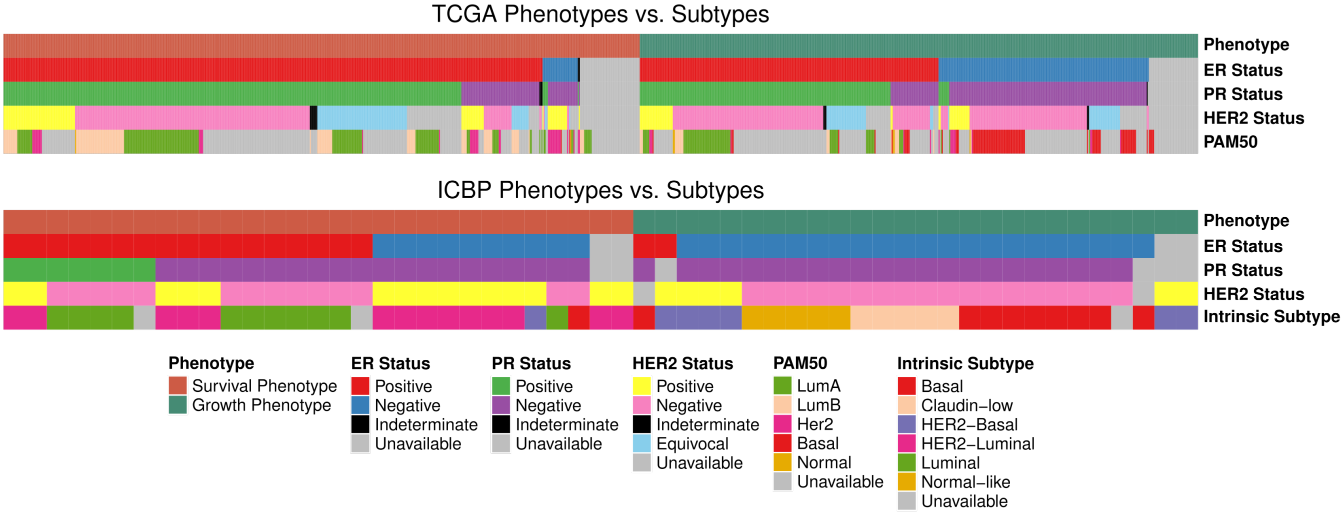

Supplemental Figure 7: Graphical representation of the IHC and intrinsic subtype status distribution for ICBP cell line and TCGA breast tumors. Each sample is represented along the X-axis and corresponding phenotype, ER, PR, HER2 and intrinsic subtype status is represented along the Y-axis. Supplemental Table 9 and 10 provides breakdown of each category, for ICBP and TCGA.

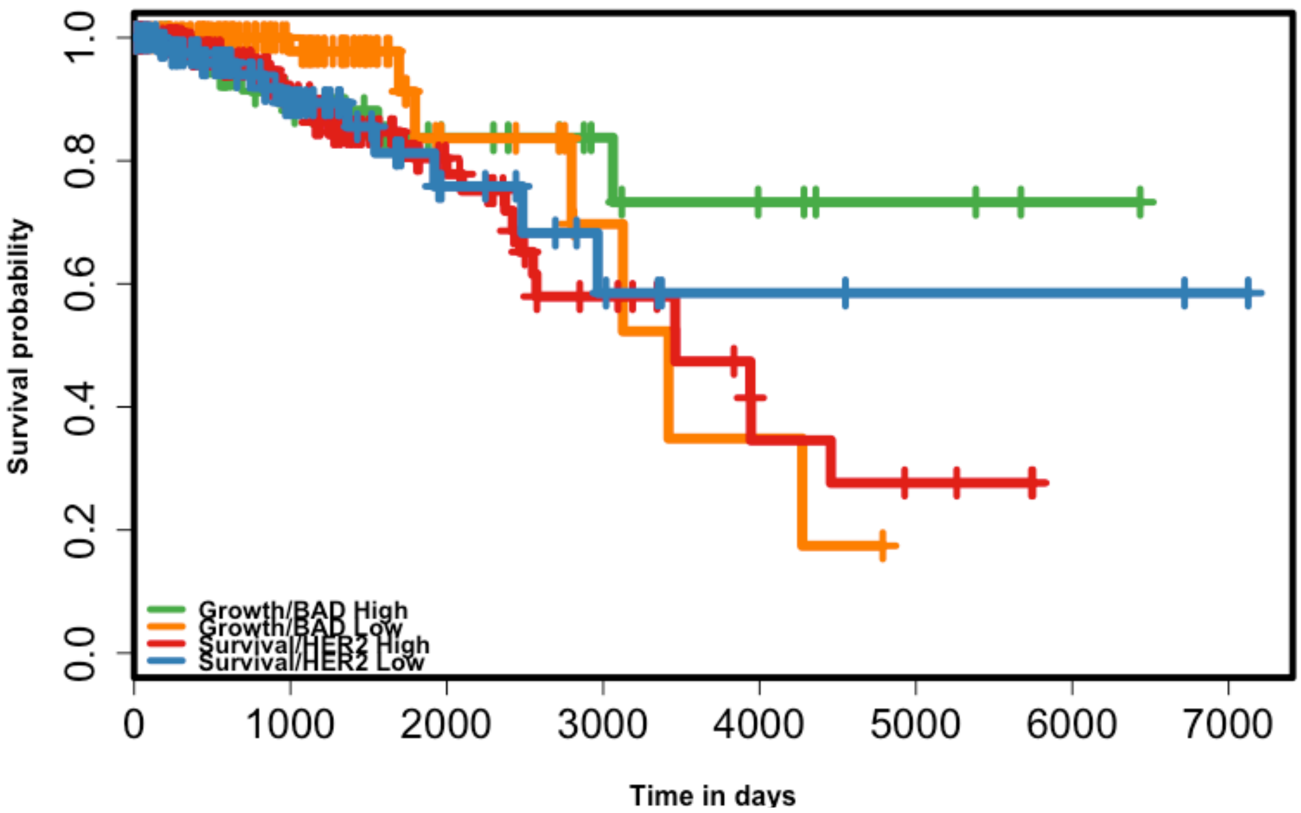

Supplemental Figure 8: Survival analysis of the four subgroups in TCGA BRCA samples (N=1119). Kaplan-Meier survival analysis for the four identified subgroups using the Peto and Peto modification of Gehan-Wilcoxon test did not show significant differences across the subgroups (λ^2=5.5, p=0.141).

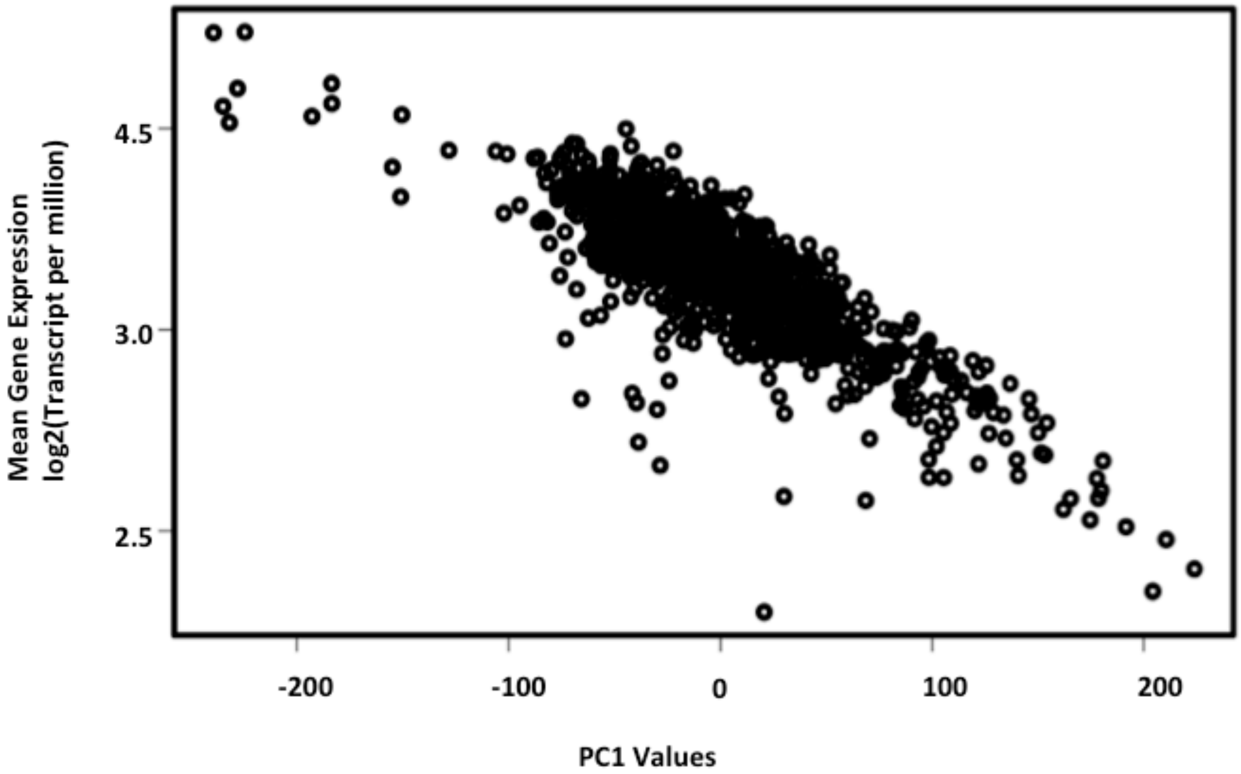

Supplemental Figure 9: Correlation between mean gene expression values for all samples and the principal component values for each sample for principal component 1 based from breast cancer (BRCA) TCGA RNA-sequencing samples (Spearman's correlations: -0.786, p-value <0.0001).

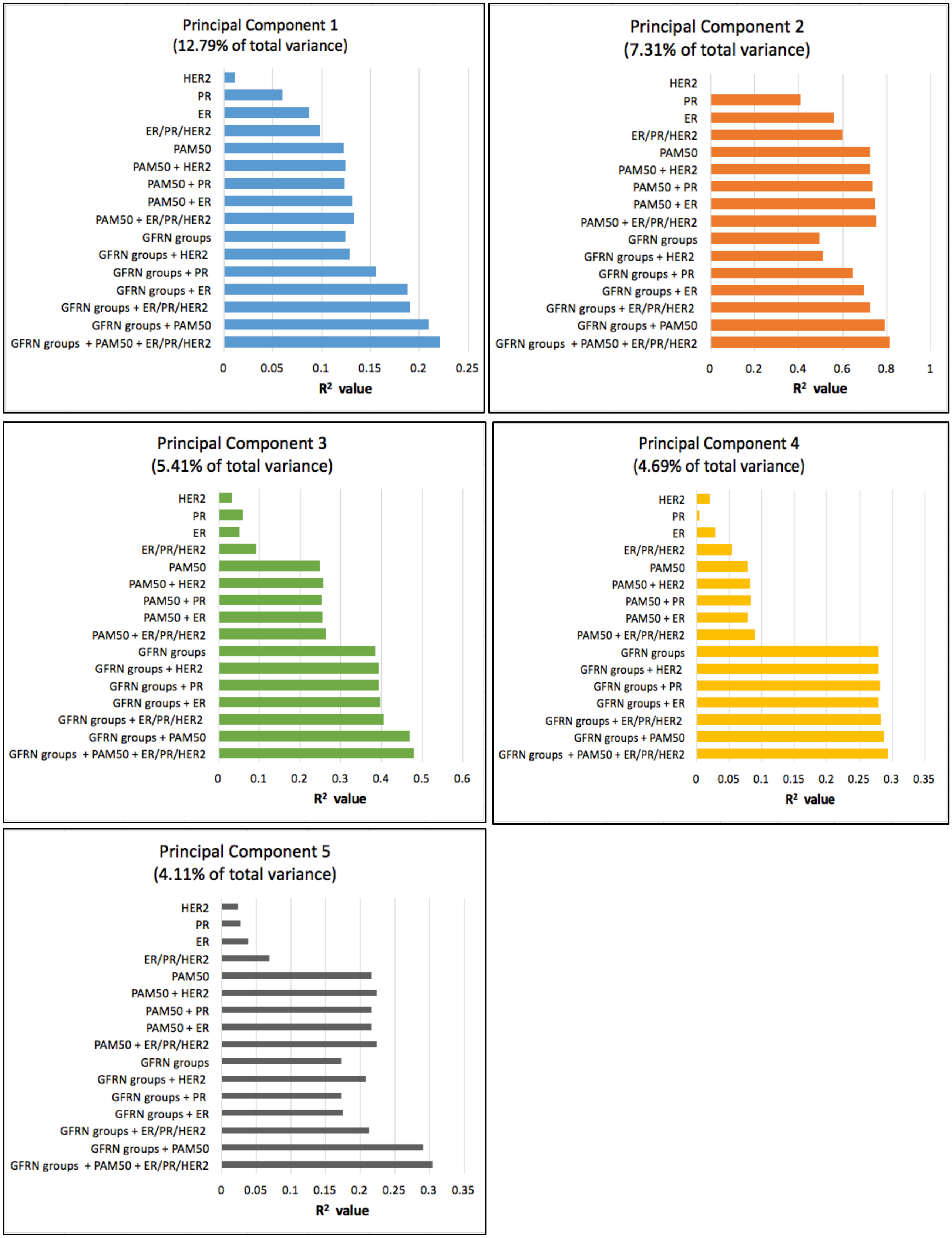

Supplemental Figure 10: Comparison of R^2 values (proportion of variance) explained by each model for principle components (PCs) 1 through 5 from TCGA RNA-sequencing breast cancer data. For each PC,

model variables include GFRN subtypes, intrinsic subtypes (PAM50), clinical subtypes (ER, ER, and HER2 status) and their combinations.

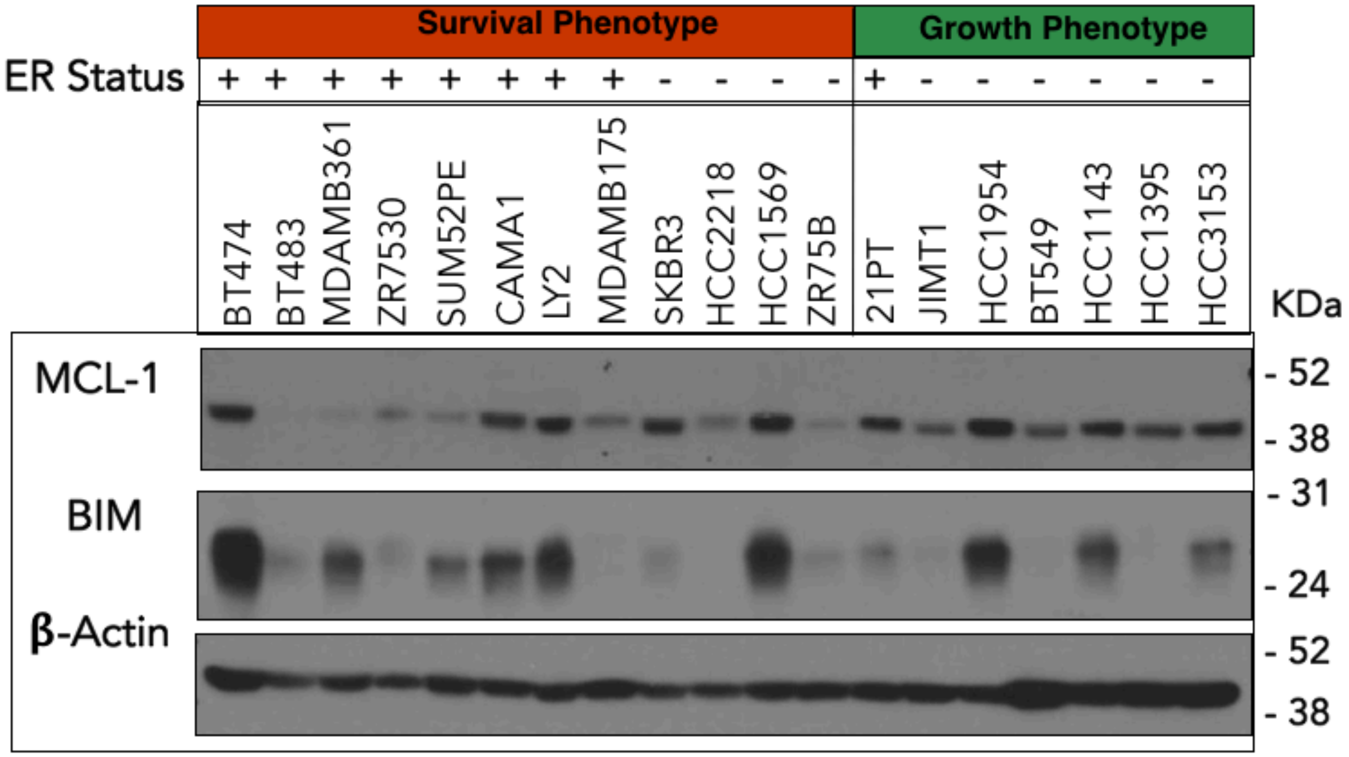

Supplemental Figure 11: Independent western blot assay for MCL-1 and BIM proteins between breast cancer cell lines from the survival and growth phenotypes. Lysates from 12 cell lines from the survival phenotype (8 ER+ and 4 ER-) and 7 cell lines from the growth phenotype (1 ER+ and 6 ER-) were probed for anti- and pro-apoptotic proteins, MCL-1 and BIM, and compared to β-actin (loading control).

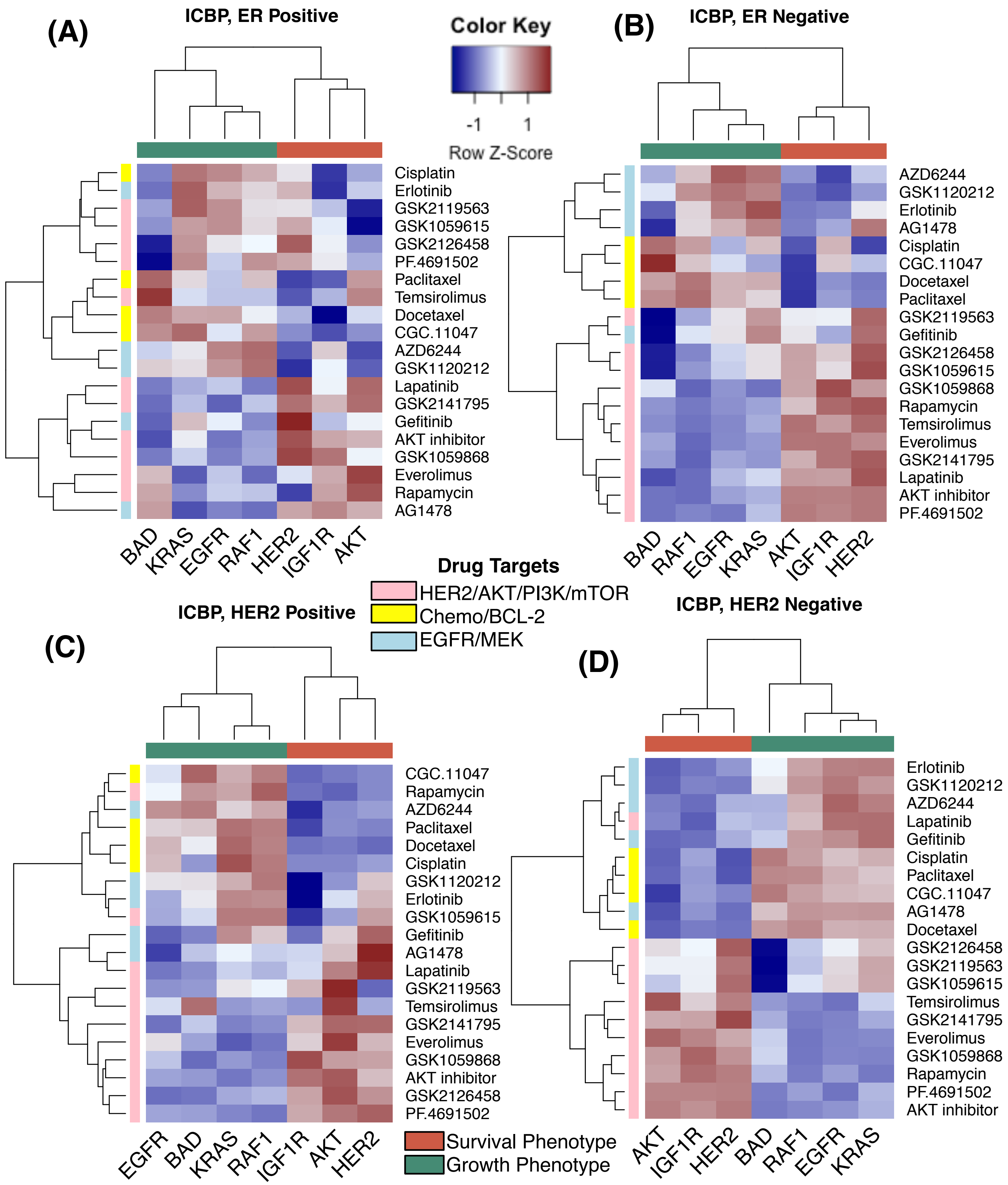

Supplemental Figure 12: Correlations between pathway activation estimates and drug response values between ER+ and ER- and between HER+ and HER2- samples in breast cancer cell lines. Colors correspond to scaled Spearman correlations between specific pathway activation estimates generated with *ASSIGN* and drug sensitivity (-logGI50) across **(A)** 18 ER+ breast cancer cell lines, **(B)** 32 ER- breast cancer cell lines from the ICBP panel, **(C)** 18 HER2+ breast cancer cell lines, and **(D)** 32

HER2- breast cancer cell lines from the ICBP panel. Red represents positive correlation and blue represents negative correlation. Pathways cluster across the x-axis as (coral color) survival phenotype and (green) growth phenotype. Drug classes are represented along the y-axis as pink (HER2/AKT/PI3K/mTOR targeted-therapies), yellow (chemotherapies/BCL-2 targeting therapies), and blue (EGFR/MEK targeted-therapies).

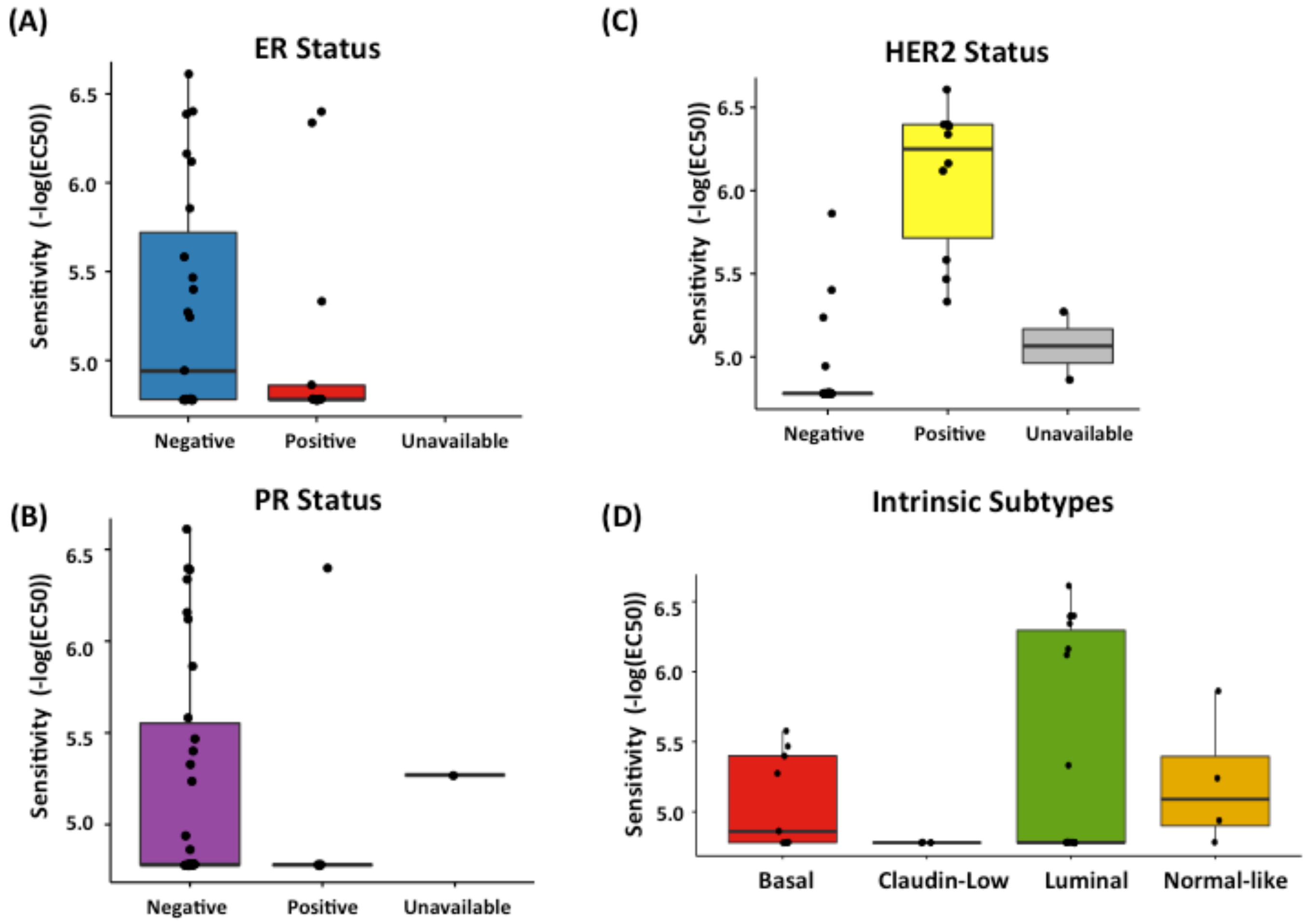

Supplemental Figure 13: Comparison of lapatinib sensitivity based on **(A)** ER status, **(B)** PR status, **(C)** HER2 status, **(D)** Intrinsic Subtypes in ICBP breast cancer cell lines. Drug sensitivity is measured in -logEC50.

Supplemental Tables

Supplemental Table 1: Spearman correlations between pathway activation estimates and proteomics data for optimum signature selection in ICBP cell line and TCGA proteomics data.

Pathway	Optimized Number of Genes	Protein	ICBP		TCGA	
			Correlation	p-value	Correlation	p-value
AKT	20	Akt	0.576	2.03E-04	0.192	1.54E-07
		PDK1	0.574	2.14E-04	0.239	5.93E-11
		PDK1_pS241	0.535	6.50E-04	0.337	5.84E-21
BAD	250	Akt	-0.456	4.33E-03	-0.150	4.43E-05
		PDK1	-0.605	8.14E-05	-0.313	4.37E-18
		PDK1_pS241	-0.518	1.02E-03	-0.232	2.23E-10
EGFR	50	EGFR	0.470	0.050	0.357	2.09E-23
		EGFR_pY1068	0.397	0.028	0.129	4.50E-04
		EGFR_pY1173	[redacted]		0.155	2.44E-05
HER2	10	HER2	0.923	0.00E+00	0.376	1.61E-05
		HER2_pY1248	0.953	0.00E+00	0.356	1.37E-04
IGF1R	100	IRS1	[redacted]		0.324	2.37E-19
		IGF1R	0.086	0.608	[redacted]	
		PDK1	0.569	2.45E-04	0.371	2.68E-25
		PDK1_pS241	0.509	1.26E-03	0.403	5.33E-30
KRAS (G12V)	200	EGFR	0.423	8.57E-03	0.493	4.05E-46
		EGFR_pY1068	0.296	7.17E-02	0.089	1.60E-02
		EGFR_pY1173	[redacted]		0.090	1.47E-02
		MEK1	[redacted]		0.116	1.69E-03
RAF	350	MEK1	0.285	0.084	0.245	1.72E-11
		PKC.alpha	0.467	3.46E-03	0.396	6.36E-29
		PKC.alpha_pS657	0.462	3.83E-03	0.415	0.00E+00

Supplemental Table 2: Top 50 gene sets predicted by GSVA between GFP (control) and HER2 overexpressing RNA-sequencing data in HMECs. Distinguishing pathways are in red.

**Hallmark + canonical (C2) gene sets (Molecular Signatures Database)**	**logFC**	**P.Value**	**adj.P.Val**
KEGG_ANTIGEN_PROCESSING_AND_PRESENTATION	-0.0207	<0.0001	<0.0001
REACTOME_IL_7_SIGNALING	-0.0239	<0.0001	<0.0001
REACTOME_CHEMOKINE_RECEPTORS_BIND_CHEMOKINES	-0.0281	<0.0001	<0.0001
BIOCARTA_CBL_PATHWAY	0.0135	<0.0001	<0.0001
BIOCARTA_COMP_PATHWAY	-0.0395	<0.0001	<0.0001
PID_VEGFR1_PATHWAY	0.0087	<0.0001	<0.0001
ST_G_ALPHA_S_PATHWAY	-0.0150	<0.0001	<0.0001
BIOCARTA_EPONFKB_PATHWAY	-0.0176	<0.0001	<0.0001
REACTOME_CELL_EXTRACELLULAR_MATRIX_INTERACTIONS	0.0180	<0.0001	<0.0001
BIOCARTA_RB_PATHWAY	0.0161	<0.0001	0.0001
BIOCARTA_IL22BP_PATHWAY	-0.0170	<0.0001	0.0001
BIOCARTA_IL10_PATHWAY	-0.0125	<0.0001	0.0001
BIOCARTA_P53HYPOXIA_PATHWAY	-0.0094	<0.0001	0.0001
KEGG_PATHOGENIC_ESCHERICHIA_COLI_INFECTION	0.0067	<0.0001	0.0001
REACTOME_REGULATION_OF_IFNA_SIGNALING	-0.0127	<0.0001	0.0002
KEGG_OOCYTE_MEIOSIS	0.0086	<0.0001	0.0003
REACTOME_RECYCLING_PATHWAY_OF_L1	0.0107	<0.0001	0.0003
BIOCARTA_SPRY_PATHWAY	0.0094	<0.0001	0.0003
KEGG_FOCAL_ADHESION	0.0067	<0.0001	0.0003
BIOCARTA_IL7_PATHWAY	-0.0102	<0.0001	0.0003
PID_REELINPATHWAY	0.0082	<0.0001	0.0003
KEGG_GAP_JUNCTION	0.0075	<0.0001	0.0004
PID_ILK_PATHWAY	0.0105	<0.0001	0.0005
REACTOME_SEMAPHORIN_INTERACTIONS	0.0079	<0.0001	0.0005
PID_NECTIN_PATHWAY	**0.0055**	**<0.0001**	**0.0006**
REACTOME_SIGNALING_BY_RHO_GTPASES	0.0061	<0.0001	0.0006
REACTOME_PEPTIDE_LIGAND_BINDING_RECEPTORS	-0.0108	<0.0001	0.0006
PID_INTEGRIN_A9B1_PATHWAY	0.0169	<0.0001	0.0007
REACTOME_KINESINS	0.0158	<0.0001	0.0007
KEGG_SELENOAMINO_ACID_METABOLISM	-0.0088	<0.0001	0.0007
PID_INTEGRIN_A4B1_PATHWAY	0.0100	<0.0001	0.0008
REACTOME_PLATELET_HOMEOSTASIS	0.0071	<0.0001	0.0008
**REACTOME_GRB2_EVENTS_IN_ERBB2_SIGNALING**	0.0118	<0.0001	0.0008
KEGG_GLYCOSPHINGOLIPID_BIOSYNTHESIS_LACTO_AND_NEOLACTO_SERIES	0.0116	<0.0001	0.0008
REACTOME_G0_AND_EARLY_G1	0.0132	<0.0001	0.0008
BIOCARTA_CELLCYCLE_PATHWAY	0.0132	<0.0001	0.0008
PID_AURORA_A_PATHWAY	0.0106	<0.0001	0.0008
PID_S1P_S1P1_PATHWAY	0.0075	<0.0001	0.0009
HALLMARK_GLYCOLYSIS	0.0063	<0.0001	0.0009
HALLMARK_INTERFERON_GAMMA_RESPONSE	-0.0225	<0.0001	0.0009
REACTOME_P75NTR_RECRUITS_SIGNALLING_COMPLEXES	0.0093	<0.0001	0.0009
**PID_ERBB_NETWORK_PATHWAY**	**0.0147**	<0.0001	**0.0009**
KEGG_CALCIUM_SIGNALING_PATHWAY	0.0055	<0.0001	0.0009
REACTOME_SIGNALING_BY_FGFR1_FUSION_MUTANTS	-0.0083	<0.0001	0.0009
BIOCARTA_NO1_PATHWAY	0.0086	<0.0001	0.0009
REACTOME_METABOLISM_OF_POLYAMINES	0.0109	<0.0001	0.0010
KEGG_AMINO_SUGAR_AND_NUCLEOTIDE_SUGAR_METABOLISM	0.0088	<0.0001	0.0010
REACTOME_BOTULINUM_NEUROTOXICITY	0.0156	<0.0001	0.0010
REACTOME_REGULATION_OF_COMPLEMENT_CASCADE	-0.0235	<0.0001	0.0010

Supplemental Table 3: Top 50 gene sets predicted by GSVA between GFP (control) and IGF1R overexpressing RNA-sequencing data in HMECs. Distinguishing pathways are in red..

**Hallmark + canonical (C2) gene sets (Molecular Signatures Database)**	**logFC**	**P.Value**	**adj.P.Val**
REACTOME_AMINO_ACID_SYNTHESIS_AND_INTERCONVERSION_TRANSAMINATION	0.064	<0.0001	<0.0001
KEGG_AMINO_SUGAR_AND_NUCLEOTIDE_SUGAR_METABOLISM	0.029	<0.0001	<0.0001
REACTOME_DIABETES_PATHWAYS	0.027	<0.0001	<0.0001
PID_ATF2_PATHWAY	0.028	<0.0001	<0.0001
REACTOME_UNFOLDED_PROTEIN_RESPONSE	0.036	<0.0001	<0.0001
REACTOME_IL_6_SIGNALING	0.045	<0.0001	<0.0001
REACTOME_ACTIVATION_OF_CHAPERONE_GENES_BY_XBP1S	0.030	<0.0001	<0.0001
HALLMARK_UNFOLDED_PROTEIN_RESPONSE	0.028	<0.0001	<0.0001
REACTOME_ACTIVATION_OF_GENES_BY_ATF4	0.053	<0.0001	<0.0001
PID_IL23PATHWAY	0.029	<0.0001	<0.0001
REACTOME_PERK_REGULATED_GENE_EXPRESSION	0.050	<0.0001	<0.0001
REACTOME_SYNTHESIS_OF_SUBSTRATES_IN_N_GLYCAN_BIOSYTHESIS	0.044	<0.0001	<0.0001
KEGG_GLYCINE_SERINE_AND_THREONINE_METABOLISM	0.034	<0.0001	<0.0001
REACTOME_ACTIVATION_OF_CHAPERONES_BY_ATF6_ALPHA	0.028	<0.0001	<0.0001
HALLMARK_CHOLESTEROL_HOMEOSTASIS	0.019	<0.0001	<0.0001
KEGG_ANTIGEN_PROCESSING_AND_PRESENTATION	-0.017	<0.0001	<0.0001
**HALLMARK_MTORC1_SIGNALING**	0.019	<0.0001	<0.0001
KEGG_NITROGEN_METABOLISM	0.022	<0.0001	<0.0001
BIOCARTA_CYTOKINE_PATHWAY	0.050	<0.0001	<0.0001
BIOCARTA_GRANULOCYTES_PATHWAY	0.057	<0.0001	<0.0001
REACTOME_SYNTHESIS_SECRETION_AND_INACTIVATION_OF_GIP	0.022	<0.0001	<0.0001
ST_STAT3_PATHWAY	0.022	<0.0001	<0.0001
KEGG_PROTEIN_EXPORT	0.016	<0.0001	<0.0001
KEGG_ALANINE_ASPARTATE_AND_GLUTAMATE_METABOLISM	0.022	<0.0001	<0.0001
KEGG_FRUCTOSE_AND_MANNOSE_METABOLISM	0.015	<0.0001	<0.0001
REACTOME_GLUCONEOGENESIS	0.016	<0.0001	<0.0001
REACTOME_BASIGIN_INTERACTIONS	0.017	<0.0001	<0.0001
PID_REG_GR_PATHWAY	0.012	<0.0001	<0.0001
BIOCARTA_ERYTH_PATHWAY	0.028	<0.0001	<0.0001
BIOCARTA_IL10_PATHWAY	0.013	<0.0001	<0.0001
REACTOME_BIOSYNTHESIS_OF_THE_N_GLYCAN_PRECURSOR_DOLICHOL_LIPID	0.029	<0.0001	<0.0001
PID_AP1_PATHWAY	0.019	<0.0001	<0.0001
KEGG_NOD_LIKE_RECEPTOR_SIGNALING_PATHWAY	0.011	<0.0001	<0.0001
PID_NECTIN_PATHWAY	-0.007	<0.0001	<0.0001
PID_P38ALPHABETADOWNSTREAMPATHWAY	0.016	<0.0001	<0.0001
BIOCARTA_TEL_PATHWAY	0.009	<0.0001	<0.0001
BIOCARTA_LAIR_PATHWAY	0.060	<0.0001	<0.0001
**BIOCARTA_IGF1MTOR_PATHWAY**	0.014	<0.0001	<0.0001
REACTOME_CIRCADIAN_CLOCK	0.008	<0.0001	<0.0001
REACTOME_BMAL1_CLOCK_NPAS2_ACTIVATES_CIRCADIAN_EXPRESSION	0.010	<0.0001	<0.0001
BIOCARTA_IL6_PATHWAY	0.012	<0.0001	<0.0001
REACTOME_INCRETIN_SYNTHESIS_SECRETION_AND_INACTIVATION	0.014	<0.0001	<0.0001
REACTOME_PLATELET_ADHESION_TO_EXPOSED_COLLAGEN	0.016	<0.0001	<0.0001
BIOCARTA_LYM_PATHWAY	0.047	<0.0001	<0.0001
HALLMARK_GLYCOLYSIS	0.008	<0.0001	<0.0001
PID_CDC42_REG_PATHWAY	-0.010	<0.0001	<0.0001
BIOCARTA_TALL1_PATHWAY	-0.013	<0.0001	<0.0001
REACTOME_ASSOCIATION_OF_LICENSING_FACTORS_WITH_THE_PRE_REPLICT…	0.024	<0.0001	<0.0001
REACTOME_CYTOSOLIC_TRNA_AMINOACYLATION	0.018	<0.0001	<0.0001

Supplemental Table 4: Top 50 gene sets predicted by GSVA between GFP (control) and AKT1 overexpressing RNA-sequencing data in HMECs. Expected pathways are in red.

Hallmark + canonical (C2) gene sets (Molecular Signatures Database)	logFC	P.Value	adj.P.Val
REACTOME_CHEMOKINE_RECEPTORS_BIND_CHEMOKINES	-0.028	<0.0001	<0.0001
REACTOME_REVERSIBLE_HYDRATION_OF_CARBON_DIOXIDE	0.035	<0.0001	<0.0001
BIOCARTA_RB_PATHWAY	-0.021	<0.0001	<0.0001
KEGG_FRUCTOSE_AND_MANNOSE_METABOLISM	0.015	<0.0001	<0.0001
REACTOME_GLYCOLYSIS	0.011	<0.0001	<0.0001
REACTOME_SIGNALING_BY_BMP	-0.015	<0.0001	<0.0001
REACTOME_DOWNREGULATION_OF_SMAD2_3_SMAD4_TRANSCRIPTIONAL…	-0.015	<0.0001	<0.0001
REACTOME_TRANSCRIPTIONAL_ACTIVITY_OF_SMAD2_SMAD3_SMA…	-0.009	<0.0001	<0.0001
PID_SYNDECAN_2_PATHWAY	-0.009	<0.0001	<0.0001
PID_NECTIN_PATHWAY	-0.007	<0.0001	<0.0001
REACTOME_YAP1_AND_WWTR1_TAZ_STIMULATED_GENE_EXPRESSION	-0.010	<0.0001	<0.0001
REACTOME_SIGNAL_ATTENUATION	-0.014	<0.0001	<0.0001
REACTOME_GLUCOSE_METABOLISM	0.008	<0.0001	<0.0001
PID_RHOA_PATHWAY	-0.008	<0.0001	<0.0001
BIOCARTA_P53_PATHWAY	-0.011	<0.0001	<0.0001
PID_P53DOWNSTREAMPATHWAY	-0.007	<0.0001	<0.0001
REACTOME_FORMATION_OF_TUBULIN_FOLDING_INTERMEDIATES_BY_CC…	0.007	<0.0001	0.0001
REACTOME_RNA_POL_I_RNA_POL_III_AND_MITOCHONDRIAL_TRANSCRIPTION	0.008	<0.0001	0.0001
REACTOME_PECAM1_INTERACTIONS	-0.013	<0.0001	0.0001
HALLMARK_WNT_BETA_CATENIN_SIGNALING	-0.008	<0.0001	0.0001
KEGG_PENTOSE_PHOSPHATE_PATHWAY	0.013	<0.0001	0.0001
BIOCARTA_CBL_PATHWAY	-0.010	<0.0001	0.0002
BIOCARTA_AMI_PATHWAY	-0.018	<0.0001	0.0002
HALLMARK_TNFA_SIGNALING_VIA_NFKB	-0.011	<0.0001	0.0002
REACTOME_REGULATION_OF_GENE_EXPRESSION_IN_BETA_CELLS	0.014	<0.0001	0.0003
REACTOME_CELL_EXTRACELLULAR_MATRIX_INTERACTIONS	-0.011	<0.0001	0.0003
REACTOME_BILE_SALT_AND_ORGANIC_ANION_SLC_TRANSPORTERS	0.018	<0.0001	0.0003
REACTOME_ZINC_TRANSPORTERS	0.011	<0.0001	0.0003
BIOCARTA_NTHI_PATHWAY	-0.011	<0.0001	0.0004
PID_REG_GR_PATHWAY	-0.008	<0.0001	0.0004
KEGG_HOMOLOGOUS_RECOMBINATION	-0.011	<0.0001	0.0004
PID_HIF1_TFPATHWAY	0.010	<0.0001	0.0004
REACTOME_GLUCONEOGENESIS	0.010	<0.0001	0.0004
BIOCARTA_DNAFRAGMENT_PATHWAY	-0.021	<0.0001	0.0004
BIOCARTA_DC_PATHWAY	-0.033	<0.0001	0.0004
BIOCARTA_ECM_PATHWAY	-0.007	<0.0001	0.0004
REACTOME_RNA_POL_III_TRANSCRIPTION	0.009	<0.0001	0.0004
**REACTOME_DOWNREGULATION_OF_ERBB2_ERBB3_SIGNALING**	0.011	<0.0001	0.0004
REACTOME_P75NTR_RECRUITS_SIGNALLING_COMPLEXES	-0.010	<0.0001	0.0004
BIOCARTA_GRANULOCYTES_PATHWAY	-0.030	<0.0001	0.0005
BIOCARTA_ARAP_PATHWAY	-0.011	<0.0001	0.0005
REACTOME_FACTORS_INVOLVED_IN_MEGAKARYOCYTE_DEVELOPMENT…	-0.008	<0.0001	0.0005
REACTOME_REGULATION_OF_RHEB_GTPASE_ACTIVITY_BY_AMPK	-0.014	<0.0001	0.0005
HALLMARK_IL6_JAK_STAT3_SIGNALING	-0.008	<0.0001	0.0005
PID_TOLL_ENDOGENOUS_PATHWAY	-0.012	<0.0001	0.0006
REACTOME_HS_GAG_BIOSYNTHESIS	-0.007	<0.0001	0.0006
REACTOME_RECYCLING_PATHWAY_OF_L1	-0.008	<0.0001	0.0006
PID_FAK_PATHWAY	-0.008	<0.0001	0.0006
BIOCARTA_ARENRF2_PATHWAY	-0.013	<0.0001	0.0007

Supplemental Table 5: Top 50 gene sets predicted by GSVA between GFP (control) and BAD overexpressing RNA-sequencing data in HMECs Expected pathways are in red.

Hallmark + canonical (C2) gene sets (Molecular Signatures Database)	logFC	P.Value	adj.P.Val
REACTOME_CHEMOKINE_RECEPTORS_BIND_CHEMOKINES	-0.055	<0.0001	<0.0001
KEGG_ANTIGEN_PROCESSING_AND_PRESENTATION	-0.031	<0.0001	<0.0001
BIOCARTA_INFLAM_PATHWAY	-0.043	<0.0001	<0.0001
BIOCARTA_NTHI_PATHWAY	-0.030	<0.0001	<0.0001
**PID_FRA_PATHWAY**	-0.030	<0.0001	<0.0001
PID_SYNDECAN_2_PATHWAY	-0.017	<0.0001	<0.0001
PID_ATF2_PATHWAY	-0.020	<0.0001	<0.0001
BIOCARTA_P53HYPOXIA_PATHWAY	-0.019	<0.0001	<0.0001
BIOCARTA_TID_PATHWAY	-0.029	<0.0001	<0.0001
PID_SYNDECAN_3_PATHWAY	-0.033	<0.0001	<0.0001
BIOCARTA_PPARA_PATHWAY	-0.017	<0.0001	<0.0001
HALLMARK_TNFA_SIGNALING_VIA_NFKB	-0.026	<0.0001	<0.0001
PID_REG_GR_PATHWAY	-0.017	<0.0001	<0.0001
BIOCARTA_IL7_PATHWAY	-0.020	<0.0001	<0.0001
BIOCARTA_FREE_PATHWAY	-0.032	<0.0001	<0.0001
BIOCARTA_IL10_PATHWAY	-0.019	<0.0001	<0.0001
PID_AP1_PATHWAY	-0.027	<0.0001	<0.0001
REACTOME_PEPTIDE_LIGAND_BINDING_RECEPTORS	-0.020	<0.0001	<0.0001
BIOCARTA_STEM_PATHWAY	-0.078	<0.0001	<0.0001
BIOCARTA_IL17_PATHWAY	-0.061	<0.0001	<0.0001
KEGG_CYTOKINE_CYTOKINE_RECEPTOR_INTERACTION	-0.018	<0.0001	<0.0001
PID_RHOA_PATHWAY	-0.013	<0.0001	<0.0001
PID_IL8CXCR1_PATHWAY	-0.023	<0.0001	<0.0001
BIOCARTA_ARENRF2_PATHWAY	-0.028	<0.0001	<0.0001
BIOCARTA_GRANULOCYTES_PATHWAY	-0.061	<0.0001	<0.0001
PID_NFAT_TFPATHWAY	-0.030	<0.0001	<0.0001
BIOCARTA_CYTOKINE_PATHWAY	-0.053	<0.0001	<0.0001
BIOCARTA_ERYTH_PATHWAY	-0.028	<0.0001	<0.0001
BILD_HRAS_ONCOGENIC_SIGNATURE	-0.021	<0.0001	<0.0001
PID_IL23PATHWAY	-0.015	<0.0001	<0.0001
REACTOME_RNA_POL_III_CHAIN_ELONGATION	0.023	<0.0001	<0.0001
KEGG_RNA_POLYMERASE	0.020	<0.0001	<0.0001
KEGG_EPITHELIAL_CELL_SIGNALING_IN_HELICOBACTER_PYLORI_INFECTION	-0.011	<0.0001	<0.0001
REACTOME_RNA_POL_III_TRANSCRIPTION_INITIATION_FROM_TYPE_3	0.019	<0.0001	<0.0001
BIOCARTA_IL22BP_PATHWAY	-0.022	<0.0001	<0.0001
BIOCARTA_ETS_PATHWAY	-0.022	<0.0001	<0.0001
BIOCARTA_CHEMICAL_PATHWAY	0.016	<0.0001	<0.0001
REACTOME_RNA_POL_III_TRANSCRIPTION_TERMINATION	0.020	<0.0001	<0.0001
KEGG_NOD_LIKE_RECEPTOR_SIGNALING_PATHWAY	-0.017	<0.0001	<0.0001
KEGG_PRION_DISEASES	-0.020	<0.0001	<0.0001
REACTOME_G_ALPHA_I_SIGNALLING_EVENTS	-0.013	<0.0001	<0.0001
KEGG_TOLL_LIKE_RECEPTOR_SIGNALING_PATHWAY	-0.013	<0.0001	<0.0001
PID_TAP63PATHWAY	-0.010	<0.0001	<0.0001
PID_P53DOWNSTREAMPATHWAY	-0.009	<0.0001	<0.0001
REACTOME_IL_6_SIGNALING	-0.020	<0.0001	<0.0001
HALLMARK_IL6_JAK_STAT3_SIGNALING	-0.013	<0.0001	<0.0001
KEGG_ENDOCYTOSIS	-0.014	<0.0001	<0.0001
PID_FGF_PATHWAY	-0.013	<0.0001	<0.0001
KEGG_PYRIMIDINE_METABOLISM	0.014	<0.0001	<0.0001

Supplemental Table 6. Top 50 gene sets predicted by GSVA between GFP (control) and EGFR overexpressing RNA-sequencing data in HMECs. Expected pathways are in red.

Hallmark + canonical (C2) gene sets (Molecular Signatures Database)	logFC	P.Value	adj.P.Val
REACTOME_UNWINDING_OF_DNA	0.056	<0.0001	<0.0001
REACTOME_DNA_STRAND_ELONGATION	0.044	<0.0001	<0.0001
REACTOME_ACTIVATION_OF_THE_PRE_REPLICATIVE_COMPLEX	0.045	<0.0001	<0.0001
REACTOME_CYCLIN_A_B1_ASSOCIATED_EVENTS_DURING_G2_M_TRANS…	0.027	<0.0001	<0.0001
KEGG_DNA_REPLICATION	0.033	<0.0001	<0.0001
PID_FANCONI_PATHWAY	0.026	<0.0001	<0.0001
REACTOME_G1_S_SPECIFIC_TRANSCRIPTION	0.039	<0.0001	<0.0001
REACTOME_ACTIVATION_OF_ATR_IN_RESPONSE_TO_REPLICATION_STRE..	0.041	<0.0001	<0.0001
REACTOME_G2_M_CHECKPOINTS	0.038	<0.0001	<0.0001
HALLMARK_E2F_TARGETS	0.025	<0.0001	<0.0001
PID_FOXM1PATHWAY	0.019	<0.0001	<0.0001
REACTOME_E2F_MEDIATED_REGULATION_OF_DNA_REPLICATION	0.030	<0.0001	<0.0001
PID_ATR_PATHWAY	0.026	<0.0001	<0.0001
BIOCARTA_MCM_PATHWAY	0.032	<0.0001	<0.0001
REACTOME_MITOTIC_PROMETAPHASE	0.025	<0.0001	<0.0001
REACTOME_DNA_REPLICATION	0.023	<0.0001	<0.0001
KEGG_CELL_CYCLE	0.017	<0.0001	<0.0001
HALLMARK_G2M_CHECKPOINT	0.020	<0.0001	<0.0001
REACTOME_G0_AND_EARLY_G1	0.027	<0.0001	<0.0001
REACTOME_POL_SWITCHING	0.037	<0.0001	<0.0001
REACTOME_MITOTIC_M_M_G1_PHASES	0.022	<0.0001	<0.0001
REACTOME_REPAIR_SYNTHESIS_FOR_GAP_FILLING_BY_DNA_POL_IN_TC…	0.033	<0.0001	<0.0001
REACTOME_LAGGING_STRAND_SYNTHESIS	0.036	<0.0001	<0.0001
REACTOME_CELL_CYCLE_MITOTIC	0.019	<0.0001	<0.0001
REACTOME_EXTENSION_OF_TELOMERES	0.030	<0.0001	<0.0001
KEGG_MISMATCH_REPAIR	0.028	<0.0001	<0.0001
REACTOME_SYNTHESIS_OF_DNA	0.020	<0.0001	<0.0001
REACTOME_INHIBITION_OF_REPLICATION_INITIATION_OF_DAMAGED_DNA..	0.024	<0.0001	<0.0001
BIOCARTA_MCM_PATHWAY	0.022	<0.0001	<0.0001
REACTOME_CDC6_ASSOCIATION_WITH_THE_ORC_ORIGIN_COMPLEX	0.036	<0.0001	<0.0001
PID_AURORA_B_PATHWAY	0.021	<0.0001	<0.0001
BIOCARTA_CELLCYCLE_PATHWAY	0.016	<0.0001	<0.0001
PID_PLK1_PATHWAY	0.019	<0.0001	<0.0001
REACTOME_S_PHASE	0.020	<0.0001	<0.0001
REACTOME_CELL_CYCLE	0.017	<0.0001	<0.0001
REACTOME_HOMOLOGOUS_RECOMBINATION_REPAIR_OF_REPLICATION…	0.025	<0.0001	<0.0001
PID_E2F_PATHWAY	0.015	<0.0001	<0.0001
REACTOME_MITOTIC_G1_G1_S_PHASES	0.017	<0.0001	<0.0001
REACTOME_M_G1_TRANSITION	0.019	<0.0001	<0.0001
REACTOME_KINESINS	0.019	<0.0001	<0.0001
REACTOME_G1_S_TRANSITION	0.019	<0.0001	<0.0001
REACTOME_CHROMOSOME_MAINTENANCE	0.020	<0.0001	<0.0001
REACTOME_PROCESSIVE_SYNTHESIS_ON_THE_LAGGING_STRAND	0.031	<0.0001	<0.0001
REACTOME_E2F_ENABLED_INHIBITION_OF_PRE_REPLICATION_COMPLEX…	0.029	<0.0001	<0.0001
KEGG_HOMOLOGOUS_RECOMBINATION	0.019	<0.0001	<0.0001
SA_REG_CASCADE_OF_CYCLIN_EXPR	0.024	<0.0001	<0.0001
PID_BARD1PATHWAY	0.017	<0.0001	<0.0001
REACTOME_ASSOCIATION_OF_LICENSING_FACTORS_WITH_THE_PRE_REP…	0.025	<0.0001	<0.0001
**PID_ERBB_NETWORK_PATHWAY**	0.020	<0.0001	<0.0001

Supplemental Table 7. Top 50 gene sets predicted by GSVA between GFP (control) and KRAS(G12V) overexpressing RNA-sequencing data in HMECs. Expected pathways are in red.

Hallmark + canonical (C2) gene sets (Molecular Signatures Database)	logFC	P.Value	adj.P.Val
**REACTOME_RAF_MAP_KINASE_CASCADE**	0.037	<0.0001	<0.0001
**PID_TCRRASPATHWAY**	0.044	<0.0001	<0.0001
**REACTOME_SHC1_EVENTS_IN_EGFR_SIGNALING**	0.023	<0.0001	<0.0001
**REACTOME_SHC_MEDIATED_SIGNALLING**	0.023	<0.0001	<0.0001
**REACTOME_GRB2_EVENTS_IN_ERBB2_SIGNALING**	0.032	<0.0001	<0.0001
**REACTOME_SHC1_EVENTS_IN_ERBB4_SIGNALING**	0.034	<0.0001	<0.0001
**REACTOME_SHC_RELATED_EVENTS**	0.022	<0.0001	<0.0001
BIOCARTA_P53HYPOXIA_PATHWAY	-0.030	<0.0001	<0.0001
**REACTOME_P38MAPK_EVENTS**	0.031	<0.0001	<0.0001
**REACTOME_SOS_MEDIATED_SIGNALLING**	0.026	<0.0001	<0.0001
**PID_RAS_PATHWAY**	0.020	<0.0001	<0.0001
**BILD_HRAS_ONCOGENIC_SIGNATURE**	0.019	<0.0001	<0.0001
KEGG_ANTIGEN_PROCESSING_AND_PRESENTATION	-0.037	<0.0001	<0.0001
REACTOME_IL_7_SIGNALING	-0.030	<0.0001	<0.0001
**PID_ERBB_NETWORK_PATHWAY**	0.030	<0.0001	<0.0001
**REACTOME_SIGNALLING_TO_P38_VIA_RIT_AND_RIN**	0.022	<0.0001	<0.0001
BIOCARTA_IL7_PATHWAY	-0.022	<0.0001	<0.0001
KEGG_ALDOSTERONE_REGULATED_SODIUM_REABSORPTION	0.018	<0.0001	<0.0001
BIOCARTA_TID_PATHWAY	-0.038	<0.0001	<0.0001
**PID_MAPKTRKPATHWAY**	0.022	<0.0001	<0.0001
PID_CD8TCRDOWNSTREAMPATHWAY	0.018	<0.0001	<0.0001
HALLMARK_ANGIOGENESIS	0.016	<0.0001	<0.0001
REACTOME_ARMS_MEDIATED_ACTIVATION	0.017	<0.0001	<0.0001
**BIOCARTA_SPRY_PATHWAY**	0.014	<0.0001	<0.0001
REACTOME_TIE2_SIGNALING	0.025	<0.0001	<0.0001
BIOCARTA_PPARA_PATHWAY	-0.017	<0.0001	<0.0001
**HALLMARK_KRAS_SIGNALING_UP**	0.014	<0.0001	<0.0001
REACTOME_NUCLEOTIDE_LIKE_PURINERGIC_RECEPTORS	0.029	<0.0001	<0.0001
HALLMARK_APICAL_SURFACE	0.020	<0.0001	<0.0001
KEGG_ENDOCYTOSIS	-0.010	<0.0001	<0.0001
KEGG_SPLICEOSOME	-0.018	<0.0001	<0.0001
**REACTOME_SIGNALING_BY_CONSTITUTIVELY_ACTIVE_EGFR**	0.015	<0.0001	<0.0001
REACTOME_HYALURONAN_METABOLISM	0.019	<0.0001	<0.0001
PID_ER_NONGENOMIC_PATHWAY	0.012	<0.0001	<0.0001
BIOCARTA_MAL_PATHWAY	0.019	<0.0001	<0.0001
**REACTOME_SIGNALLING_TO_RAS**	0.013	<0.0001	<0.0001
HALLMARK_IL2_STAT5_SIGNALING	0.010	<0.0001	<0.0001
BIOCARTA_TEL_PATHWAY	0.015	<0.0001	<0.0001
REACTOME_TRIGLYCERIDE_BIOSYNTHESIS	0.011	<0.0001	<0.0001
**PID_P38ALPHABETAPATHWAY**	-0.011	<0.0001	<0.0001
**REACTOME_SHC_MEDIATED_CASCADE**	0.022	<0.0001	<0.0001
BIOCARTA_EPONFKB_PATHWAY	-0.013	<0.0001	<0.0001
BIOCARTA_FIBRINOLYSIS_PATHWAY	0.037	<0.0001	<0.0001
**ST_JNK_MAPK_PATHWAY**	-0.010	<0.0001	<0.0001
**REACTOME_PROLONGED_ERK_ACTIVATION_EVENTS**	0.013	<0.0001	<0.0001
**REACTOME_GASTRIN_CREB_SIGNALLING_PATHWAY_VIA_PKC_AND_MAPK**	0.009	<0.0001	<0.0001
**PID_ERBB2ERBB3PATHWAY**	0.008	<0.0001	<0.0001
BIOCARTA_LONGEVITY_PATHWAY	-0.011	<0.0001	<0.0001

Supplemental Table 8. Top 50 gene sets predicted by GSVA between GFP (control) and RAF1 overexpressing RNA-sequencing data in HMECs. Expected pathways are in red.

Hallmark + canonical (C2) gene sets (Molecular Signatures Database)	logFC	P.Value	adj.P.Val
**BIOCARTA_SPRY_PATHWAY**	0.027	<0.0001	<0.0001
KEGG_ANTIGEN_PROCESSING_AND_PRESENTATION	-0.029	<0.0001	<0.0001
**HALLMARK_KRAS_SIGNALING_UP**	0.021	<0.0001	<0.0001
PID_REELINPATHWAY	0.018	<0.0001	<0.0001
**BIOCARTA_CBL_PATHWAY**	0.020	<0.0001	<0.0001
REACTOME_REVERSIBLE_HYDRATION_OF_CARBON_DIOXIDE	0.034	<0.0001	<0.0001
BIOCARTA_FIBRINOLYSIS_PATHWAY	0.051	<0.0001	<0.0001
PID_VEGFR1_PATHWAY	0.014	<0.0001	<0.0001
PID_INTEGRIN_A9B1_PATHWAY	0.037	<0.0001	<0.0001
BIOCARTA_SPPA_PATHWAY	0.021	<0.0001	<0.0001
BIOCARTA_IL10_PATHWAY	-0.020	<0.0001	<0.0001
PID_BMPPATHWAY	0.022	<0.0001	<0.0001
SIG_IL4RECEPTOR_IN_B_LYPHOCYTES	0.020	<0.0001	<0.0001
BIOCARTA_P53HYPOXIA_PATHWAY	-0.016	<0.0001	<0.0001
**PID_ERBB1_INTERNALIZATION_PATHWAY**	0.021	<0.0001	<0.0001
HALLMARK_TGF_BETA_SIGNALING	0.017	<0.0001	<0.0001
PID_IGF1_PATHWAY	0.015	<0.0001	<0.0001
SIG_PIP3_SIGNALING_IN_B_LYMPHOCYTES	0.013	<0.0001	<0.0001
BIOCARTA_AKAP13_PATHWAY	0.026	<0.0001	<0.0001
PID_TGFBRPATHWAY	0.012	<0.0001	<0.0001
PID_FGF_PATHWAY	0.012	<0.0001	<0.0001
REACTOME_DOWNREGULATION_OF_SMAD2_3_SMAD4_TRANS..	0.015	<0.0001	<0.0001
HALLMARK_IL2_STAT5_SIGNALING	0.011	<0.0001	<0.0001
BIOCARTA_IL22BP_PATHWAY	-0.019	<0.0001	<0.0001
KEGG_SPLICEOSOME	-0.015	<0.0001	<0.0001
SIG_BCR_SIGNALING_PATHWAY	0.013	<0.0001	<0.0001
REACTOME_SIGNAL_TRANSDUCTION_BY_L1	0.011	<0.0001	<0.0001
KEGG_ASCORBATE_AND_ALDARATE_METABOLISM	-0.019	<0.0001	<0.0001
REACTOME_RECYCLING_PATHWAY_OF_L1	0.014	<0.0001	<0.0001
REACTOME_SIGNALING_BY_TGF_BETA_RECEPTOR_COMPLEX	0.009	<0.0001	<0.0001
REACTOME_DOWNREGULATION_OF_TGF_BETA_RECEPTOR	0.013	<0.0001	<0.0001
KEGG_NATURAL_KILLER_CELL_MEDIATED_CYTOTOXICITY	0.009	<0.0001	<0.0001
KEGG_JAK_STAT_SIGNALING_PATHWAY	0.011	<0.0001	<0.0001
REACTOME_SIGNALING_BY_BMP	0.017	<0.0001	<0.0001
REACTOME_TRANSCRIPTIONAL_ACTIVITY_OF_SMAD2_SMAD3..	0.010	<0.0001	<0.0001
REACTOME_RORA_ACTIVATES_CIRCADIAN_EXPRESSION	0.012	<0.0001	<0.0001
PID_EPHRINBREVPATHWAY	0.015	<0.0001	<0.0001
REACTOME_IL_7_SIGNALING	-0.016	<0.0001	<0.0001
KEGG_VASCULAR_SMOOTH_MUSCLE_CONTRACTION	0.010	<0.0001	<0.0001
REACTOME_G_ALPHA1213_SIGNALLING_EVENTS	0.013	<0.0001	<0.0001
**PID_P38_MK2PATHWAY**	0.013	<0.0001	<0.0001
REACTOME_GAP_JUNCTION_TRAFFICKING	0.016	<0.0001	<0.0001
KEGG_FATTY_ACID_METABOLISM	-0.012	<0.0001	<0.0001
KEGG_PRION_DISEASES	-0.018	<0.0001	<0.0001
REACTOME_TGF_BETA_RECEPTOR_SIGNALING_ACTIVATES_SMADS	0.013	<0.0001	<0.0001
PID_ARF6_PATHWAY	0.011	<0.0001	<0.0001
BIOCARTA_ECM_PATHWAY	0.010	<0.0001	<0.0001
**BILD_HRAS_ONCOGENIC_SIGNATURE**	0.015	<0.0001	<0.0001
PID_CDC42_REG_PATHWAY	0.012	<0.0001	<0.0001

Supplemental Table 9: Clinical and intrinsic subtype variation within the growth and survival phenotypes in ICBP breast cancer cell lines

Subtypes	Num. in survival phenotype (N=29)	Percentage of total survival phenotype samples	Num. in growth phenotype (N=26)	Percentage of total growth phenotype samples
ER Positive	17	58.62%	1	3.84%
ER Negative	10	34.48%	22	84.62%
PR Positive	7	24.14%	0	0%
PR Negative	20	68.96%	21	80.76%
HER2 Positive	15	51.72%	2	7.69%
HER2 Negative	14	48.28%	19	73.07%
Basal	1	3.45%	9	34.62%
Claudin-low	0	0%	5	19.23%
HER2-Basal	1	3.45%	6	23.08%
HER2-Luminal	14	48.28%	0	0%
Luminal	11	37.93%	0	0%
Normal-like	0	0%	5	19.23%

Supplemental Table 10: Clinical and intrinsic subtype variation within the growth and survival phenotypes in TCGA tumor data.

**Subtypes**	**Num. in survival phenotype (N=596)**	**Percentage of total survival phenotype samples**	**Num. in growth phenotype (N=523)**	**Percentage of total growth phenotype samples**
ER Positive	505	84.73%	280	53.54%
ER Negative	33	5.54%	197	37.67%
PR Positive	435	72.99%	245	46.85%
PR Negative	102	17.11%	230	43.98%
HER2 Positive	108	18.12%	54	10.33%
HER2 Negative	251	42.11%	295	56.41%
Basal	2	0.34%	93	17.78%
HER2	41	6.88%	16	3.06%
LumA	158	26.51%	73	13.96%
LumB	106	17.79%	21	4.02%
Normal	2	0.34%	5	0.96%

Supplemental Table 11: Comparing GFRN subtypes, intrinsic subtypes (PAM50), and clinical subtypes (ER, ER, and HER2 status) in terms of contribution to principle components 1 through 5 from TCGA RNA-sequencing breast cancer data. Contributed variability from linear models are represented as R^2 values (0-1).

PC	ER (R^2)	ER + GFRN subgroups (R^2)	ER + PAM50 (R^2)
1	0.087	0.188	0.131
2	0.561	0.696	0.747
3	0.052	0.398	0.254
4	0.029	0.279	0.078
5	0.038	0.175	0.216

PC	PR	PR + GFRN subgroups	PR + PAM50
1	0.060	0.156	0.124
2	0.407	0.647	0.736
3	0.059	0.393	0.253
4	0.004	0.282	0.083
5	0.027	0.173	0.216

PC	HER2	HER2 + GFRN subgroups	HER2 + PAM50
1	0.011	0.129	0.125
2	0.000	0.509	0.725
3	0.033	0.393	0.257
4	0.021	0.279	0.082
5	0.023	0.207	0.224

PC	ER/PR/HER2	ER/PR/HER2 + GFRN subgroups	ER/PR/HER2 + PAM50
1	0.098	0.191	0.133
2	0.598	0.726	0.751
3	0.091	0.404	0.263
4	0.054	0.282	0.089
5	0.068	0.213	0.224

PC	GFRN subgroups	PAM50	GFRN subgroups + PAM50	ER/PR/HER2 + PAM50 +GFRN subgroups
1	0.124427	0.1229359	0.2100966	0.220723
2	0.4922497	0.7243437	0.7920581	0.8151674
3	0.3845233	0.2489111	0.4695138	0.4784226
4	0.2788131	0.0777884	0.2880172	0.2936144
5	0.1725182	0.2159571	0.2904661	0.3047475

Supplemental Table 12: Spearman correlations between principal component values for principal components 1-5 from TCGA BRCA gene expression data and pathway activation estimates for each oncogenic signature in TCGA BRCA gene expression data (* p-value<0.0001).

	PC 1	PC 2	PC 3	PC 4	PC 5
**AKT**	0.047	-0.572*	0.402*	0.474*	0.084
**HER2**	-0.076	-0.334*	0.366*	0.347*	-0.094
**IGF1R**	-0.284*	-0.824*	0.249*	0.358*	0.044
**EGFR**	-0.255*	0.439*	-0.538*	-0.596*	-0.266*
**RAF1**	-0.357*	0.639*	-0.434*	-0.636*	-0.347*
**KRAS**	0.108	0.762*	-0.399*	-0.443*	-0.065
**BAD**	0.401*	0.452*	0.524*	-0.139*	0.364*

Supplemental Table 13: List of cancer drugs and corresponding p-values, where GFRN phenotypes, ER, PR, or HER2 status could significantly (p-value<0.05) distinguish drug response in ICBP cell lines.

GFRN phenotype drugs	P.value	ER based drugs	P.value	PR based drugs	P.value	HER2 based drugs	P.value
AKT1/2 Inhibitor	<0.0001	AG1478	0.014	AKT1/2 Inhibitor	0.028	AG1478	0.001
AZD6244	0.007	AKT1/2 Inhibitor	0.034	Triciribine	0.001	BEZ235	0.024
CGC.11047	0.006	Bortezomib	0.041	AS.252424	0.029	BIBW2992	0.000
Erlotinib	0.012	CGC.11047	0.027	AZD6244	0.000	CPT.11	0.040
Etoposide	0.034	Erlotinib	0.001	GSK1070916	0.047	Everolimus	0.020
Everolimus	0.001	GSK461364	0.004	GSK1120212	0.000	GSK1838705	0.015
Fascaplysin	0.004	GSK2119563	0.049	GSK461364	0.001	GSK2119563	0.029
GSK1070916	0.035	MG.132	0.017	ICRF.193	0.000	GSK2126458	0.004
GSK1120212	0.003	PF.4691502	0.041	PF.3814735	0.023	GSK1059615	0.021
GSK1059868	0.018	Vorinostat	0.022	Pemetrexed	0.000	GSK650394	0.038
GSK461364	0.016	Bosutinib	0.018	VX.680	0.020	Lapatinib	>0.001
GSK2119563	0.022	Tamoxifen	0.044	ZM447439	0.010	Geldanamycin	0.021
GSK2126458	0.008	Trichostatin.A	0.048			Gefitinib	0.003
GSK2141795	0.009					NU6102	0.000
GSK650394	0.029					Olomoucine.II	0.031
Lapatinib	0.036					PF.2341066	0.005
IKK.16	0.003					PF.3814735	0.007
LBH589	0.005					Temsirolimus	0.039
MG.132	0.008					VX.680	0.019
NU6102	0.028						
PF.4691502	0.000						
Rapamycin	0.001						
Vorinostat	0.001						
Bosutinib	0.003						
Sunitinib.Malate	0.015						
Temsirolimus	0.032						
Trichostatin.A	0.000						

Supplemental Table 14: *ASSIGN* parameters used for all analyses. The default values were used for all other parameters.

**Parameter**	**Value**
adaptive_B	TRUE
adaptive_S	TRUE
mixture_beta	FALSE
S_zeroPrior	FALSE
sigma_sZero	0.05
sigma_sNonZero	0.5
iter	100,000
burn_in	50,000
